# Synthesis of two methylolanthanin derivatives and the investigation of their lanthanide and iron binding capabilities

**DOI:** 10.1039/d6dt00963h

**Published:** 2026-07-14

**Authors:** Michael W. Mertens, Sarah L. Hügel, Oliver Hagen, Sophie M. Gutenthaler-Tietze, Patrick Weis, Björn Drobot, Lena J. Daumann

**Affiliations:** a Heinrich-Heine-University Düsseldorf, Chair of Bioinorganic Chemistry Universitätsstr. 1 40225 Düsseldorf Germany Lena.Daumann@hhu.de; b Ludwig Maximilian University of Munich, Department of Chemistry Butenandtstr. 5-13 81377 Munich Germany; c Institute of Physical Chemistry, Karlsruhe Institute of Technology Fritz-Haber-Weg 2 76131 Karlsruhe Germany; d Institute of Resource Ecology Biogeochemie, Helmholtz-Zentrum Dresden-Rossendorf e.V. Bautzner Landstraße 400 01328 Dresden Germany

## Abstract

Lanthanides (Lns) have recently been recognized as essential cofactors for certain bacterial enzymes, such as methanol dehydrogenase (MDH), yet their poor bioavailability under physiological conditions has long suggested the existence of Ln-binding metallophores termed lanthanophores. In this context methylolanthanin (MLL), a chelator which is potentially involved in Ln-uptake of methylotrophic bacteria, was recently isolated and characterized. Herein we synthesized two novel MLL derivatives, *ortho*- and *meta*-MLL, for comparative studies in order to gain a deeper insight into the unusual 4-hydroxy benzoate moiety of native *para*-MLL. For this we implemented UV-vis titrations, time-resolved laser-induced fluorescence spectroscopy (TRLFS) and ion mobility spectrometry-mass spectrometry (IMS-MS) complemented by density functional theory (DFT) calculations to investigate metal-binding behavior in both solution and gas phases to trivalent Lns. As our binding studies revealed that both artificial chelators tend to precipitate Lns similar to the native *para*-MLL under biologically relevant conditions (pH: 6.0), rather than solubilizing them, the solubility products of their Eu^3+^ complexes were determined. Due to the structural resemblance of the MLL derivatives to the siderophore rhodopetrobactin B (RPB B), we also investigated iron binding. In this regard *ortho*-MLL stood out in our analysis and showed a distinctly different binding behavior from the other two derivatives.

## Abstract 2

This work presents the synthesis of two methylolanthanin derivatives and shows how the positional change of the hydroxyl group steers metal binding toward lanthanides or ferric iron.

## Graphical abstract

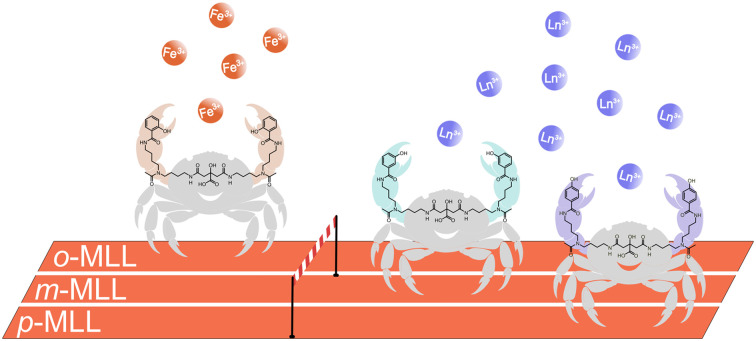

## Introduction

**Fig. 1:**
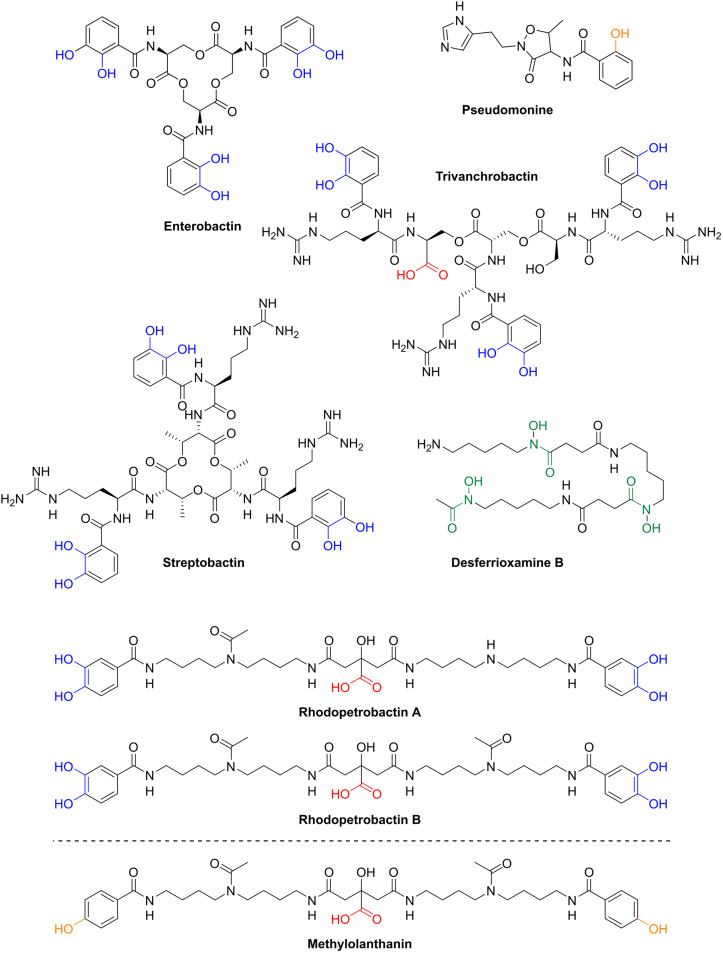
Chemical structures of representative siderophores with diverse functional groups such as phenolates (orange), catecholates (blue), hydroxamates (green) and carboxylates (red).^28–34^ The chelator methylolanthanin found in some Ln-using bacteria is included here for comparison.^35^

Modern high-tech life is dependent on the use of rare earth elements (REEs) which comprise 15 lanthanides (Lns) La–Lu and the third-row metals Sc and Y. They usually co-occur in different natural minerals such as monazite and bastnäsite^1,2^ and have long been believed to be irrelevant for living organisms due to their low bioavailability and wrongly believed scarcity.^3^ Previous reports changed this perception and showed the widespread use of Ln^3+^ instead of Ca^2+^ in active sites of methanol dehydrogenase (MDH) metalloenzymes by methylotrophic bacteria in a broad spectrum over different ecosystems.^4–8^ This development was further supported by the discovery of the first Ln-binding proteins from the lanthanome.^9–11^ Methylotrophic bacteria are able to use the C_1_-molecule methanol as their energy source,^12^ where a variety of Lns function as Lewis acids in the catalytic centre of MDH with pyrroloquinoline quinone (PQQ) as a redox active cofactor to oxidize methanol to formaldehyde.^13^ The question how these bacteria acquire Lns for their C_1_-metabolism from the environment despite their poor solubility has puzzled researchers since the beginning of this new field of research.^14,15^ Comparable to the essential trace element iron, where bacteria have developed elaborate mechanisms to acquire this metal ion despite its poor solubility under biological conditions, small chelators similar to siderophores (Greek: iron carrier), termed lanthanophores,^13,16,17^ have been suggested to be involved early on.^18^ This is not far-fetched as it has been shown that some siderophores and related molecules are also attractive chelators for Lns. For example, multiple studies involving siderophore-inspired ligand-designs such as derivatives of hydroxypyridinone (HOPO), desferrioxamine B (DFO B), enterobactin and related molecules have been shown to be excellent Ln chelators.^19–24^ This is no surprise from a coordination chemistry point of view as the trivalent Lns and ferric iron are hard Lewis acids and therefore favour hard, oxygen donor-rich ligand environments. One major difference between Ln^3+^ and Fe^3+^ is however that the former prefer to have general higher coordination numbers (CNs) of 8 or 9,^25^ in contrast to hexacoordinated Fe^3+^.^26^ Additionally, the CN can differ between early and late Lns due to the lanthanide contraction.^27^ Interestingly, among the more than 500 known siderophores (see Fig. 1 for a selected few)^28,29^ several molecules contain multiple functional groups possibly enabling the formation of eight-coordinated lanthanide complexes (*e.g.* trivanchrobactin^30^ or streptobactin^31^).

Furthermore, it is not uncommon that some bacteria produce multiple siderophores with strong structural similarity. This phenomenon can be found in *Rhodopseudomonas palustris* str. CGA009, which secretes the chelators RPB A and RPB B, structurally only differing by an additional acetyl moiety.^32^ Taking all these aspects into account, one could hypothesize that a similar diversity also exists for potential lanthanophores or that some bacteria might even use the same chelator or modifications thereof for Fe^3+^ and Ln^3+^ uptake.

Some hints of secreted lanthanophores have been observed in the literature.^36^ For example, the presence of a Ln-binding metallophore was indicated in the spent medium of *Methylobacterium aquaticum* Strain 22A, which is hypothesized to synthesize a staphyloferrin B-like siderophore, as the spent medium was able to solubilize poorly soluble Ln_2_O_3_.^36^ Furthermore, a metallophore termed methylolanthanin (MLL), which is potentially involved in Ln-uptake of methylotrophic bacteria, was recently isolated and characterized from the spent medium of *Methylobacterium extorquens* (*M. extorquens*) AM1.^35^ Surprisingly, *in vitro* studies have shown precipitation of MLL upon addition of Lns under a biologically relevant pH of 6.0 and nanomolar concentrations. This behavior is in contrast to the general expectations that metallophores solubilize metals.^37^ However, this finding does not necessarily exclude that MLL may still be involved in Ln-uptake in an *in vivo* context or function, for example, as a sensor molecule. Furthermore, a complex regulatory interplay between the Fe^3+^ and Ln^3+^ metabolism seems to exist. When *M. extorquens* AM1 is grown under iron limiting conditions, MLL production is significantly upregulated, suggesting an extended role beyond simple Ln acquisition to a broader involvement in metal homeostasis.^37^ Additionally, the cells grown under iron limiting conditions showed a vast increase in neodymium accumulation and an upregulation of the *mll* promoter activity.^35,37^ When MLL was initially isolated as a chelator that is involved in Ln-metabolism, it was surprising that it effectively contained fewer donor atoms than the structurally related siderophore RPB B (see Fig. 1). Given the higher coordination number preference of Ln^3+^ over Fe^3+^ one would have expected to find a chelator that provides an octadentate environment. In addition, the 4-hydroxy benzoate moiety is also unusual, as the hydroxyl group in phenolic siderophores is typically located at the *ortho*-position as in pseudomonine (see Fig. 1)^34^ and has not been reported previously at the *para*-position for phenolic siderophores.^38^ This prompted us to change the position of this functional group synthetically, yielding two novel MLL derivatives in order to examine the impact of the hydroxyl group positioning on Ln^3+^ and Fe^3+^ binding.

## Results and discussion

### Synthesis of *meta*-MLL and *ortho*-MLL

**Fig. 2:**
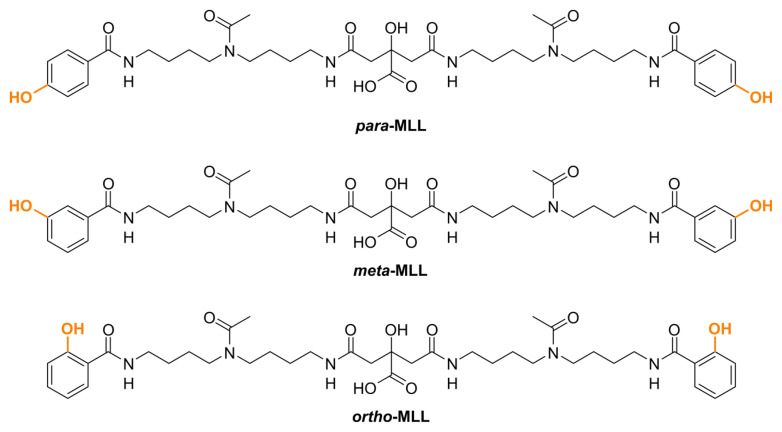
Natural chelator *para*-MLL and its derivatives *meta*- and *ortho*-MLL. The hydroxy groups at the aromatic rings are highlighted in orange.

To investigate the influence of the hydroxy-group positioning within the hydroxy benzoate moiety on the metal-binding capabilities of the natural metallophore *para*-MLL, we synthesized two different derivatives: *meta*- and *ortho*-MLL (Fig. 2).

**Scheme 1:**
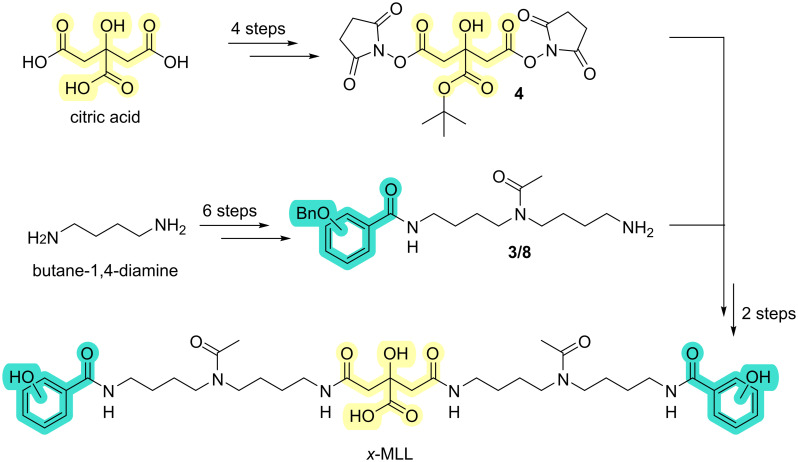
Synthetic pathway toward the MLL derivatives starting from citric acid and butane-1,4-diamine. The citric acid core is highlighted in yellow and the position of the different hydroxyl groups in turquoise. The benzyl protected hydroxyl moiety at the aromatic ring is abbreviated as BnO.

For this, we used the previously published synthetic routes for the syntheses of RPB B and *para*-MLL,^37^ which were originally based on the petrobactin synthesis of Gardner *et al.*^39^ as starting point (Scheme 1). The initial steps involved the selective *tert*-butyloxycarbonyl (Boc) protection of one amino group of butane-1,4-diamine and subsequent reaction of the remaining unprotected amine with 4-bromobutyronitrile. The secondary amine group was acetylated with acetyl chloride followed by the reduction of the nitrile group using a combination of sodium borohydride and cobalt dichloride hexahydrate, based on the work by Bentz *et al.*^40^ Completion of the side chain was achieved by the addition of the aromatic building block. In this procedure the position of the hydroxyl group was varied by using the respective (benzyloxy)benzoic acid depending on the desired chelator. For the natural chelator *para*-MLL 4-(benzyloxy)benzoic acid, for *meta*-MLL 3-(benzyloxy)benzoic acid and for *ortho*-MLL 2-(benzyloxy)benzoic acid were used. Therefore, the benzoic acid derivative (turquoise part in Scheme 1) was activated with oxalyl dichloride and reacted under basic conditions with the amine moiety, followed by Boc-deprotection of the secondary amine using trifluoracetic acid, yielding compound 3 or 8. In a three-step synthesis the citric acid core was protected by a *tert*-butyl group at the central carboxyl moiety prior to the coupling to ensure the addition of only two side chains to the citric acid core. In order to prevent decarboxylation of the central carboxylic acid group caused by UV exposure, the following steps were carried out under the exclusion of light.^41^ The coupling reaction was performed with *N*,*N*′-dicyclohexylcarbodiimide (DCC) and *N*-hydroxysuccinimide (NHS) to obtain the *O*-benzyl-protected MLL derivative.

The last step involved cleavage of the protection groups with concentrated hydrochloric acid. As citric acid based siderophores have been reported to undergo cyclization under acidic conditions forming an imide structure as a decomposition product,^42,43^ minor adjustments during the synthesis of each derivative were made to improve the yield of the previously reported 13% for *para*-MLL.^37^ In the synthesis of *meta*-MLL the reaction was closely followed by liquid chromatography-mass spectrometry (LC-MS) and stopped, when the first imide formation appeared, reducing the reaction time from 3 d to 2 h. To further minimize imide formation for *ortho*-MLL the hydrochloric acid was neutralized after completion of the reaction using solutions of saturated sodium hydrogen carbonate and 6 M sodium hydroxide prior to the removal of the solvent under reduced pressure. The reduction of the reaction time had the biggest impact and improved the yield by roughly 30% and the neutralization step by an additional 5%. Both chelators were obtained after preparative HPLC purification leading to the desired products *meta*-MLL and *ortho*-MLL (Fig. 2) with yields of 41% and 46%, respectively. These yields are comparable to those reported in the literature for similar syntheses following a deprotection protocol *via* hydrogenation catalyzed by palladium on charcoal,^39^ with the advantage of having a shorter reaction time and fewer reaction steps.

### Lanthanide binding studies *via* UV-vis

**Fig. 3:**
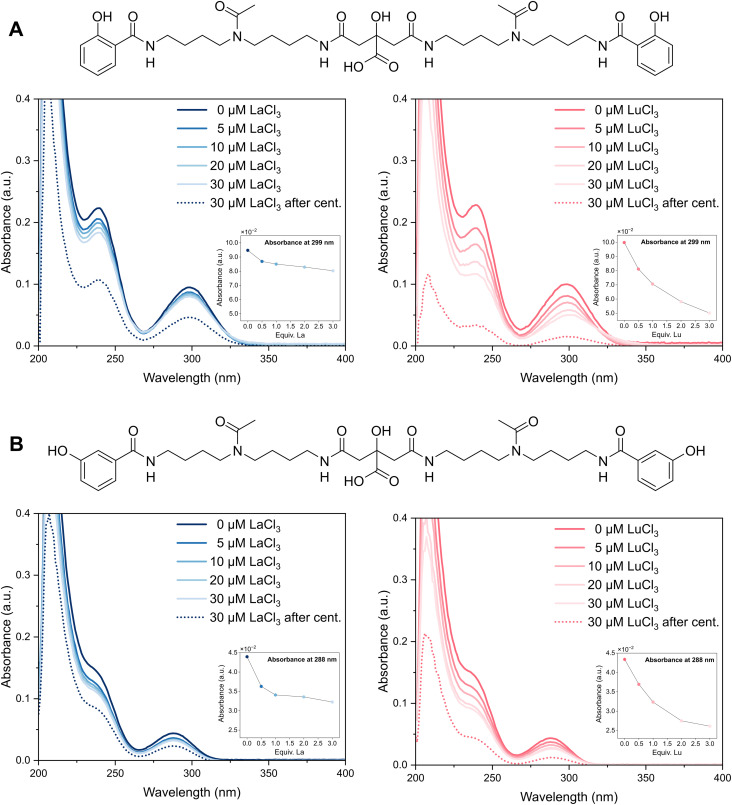
UV-vis spectra of LnCl_3_ (Ln = La, Lu) *vs x*-MLL (10 µM) titrations in buffer (10 mM MOPSO, 100 mM KCl, pH 6.0). Titrations were performed over four steps with final Ln^3+^ concentrations ranging from 0 μM to 30 μM (0.0 to 3.0 equiv. Ln^3+^). The 30 µM samples were remeasured after centrifugation (21 000*g*, 15 minutes, 25 °C). **A** represents the data for *ortho*-MLL and **B** for *meta*-MLL. The insets show the absorbance at the maximum of the respective chelator *versus* the added equivalents of Ln^3+^ without the centrifugation step.

Curious to find out whether the structurally modified MLL derivatives would exhibit a different binding behavior toward Lns, we started our investigations with UV-vis titration experiments. For better comparison with the literature-known *para*-MLL, we used a previously reported setup.^37^ Due to the decreasing ionic radii over the Ln series caused by the lanthanide contraction, we chose four different lanthanides (La^3+^, Nd^3+^, Eu^3+^, and Lu^3+^) for this experiment to showcase the variety of different ionic radii. In the case of Ln binding, we expected that the addition of the metal would result in spectroscopic changes such as the appearance of new bands or shifts of the existing ones. The UV-vis titration experimental data for the two new derivatives with La^3+^ and Lu^3+^ are shown in Fig. 3; for Eu^3+^ and Nd^3+^ refer to Fig. S3 (SI). The MLL derivatives exhibit multiple local maxima and bear distant resemblance to the UV-vis data of 3,4-catechol ligands reported in the literature, with local maxima at 255 and 294 nm.^32^ For the *ortho* derivative a distinct shoulder at 239 and a maximum at 299 nm are found, which reveal that it is the spectroscopically most red-shifted of all MLLs. *meta*-MLL in contrast shows a small shoulder at 237 nm and an absorption maximum at 288 nm. After the addition of the respective lanthanide chloride, we observed an equal decrease in absorbance with an increase of the Ln concentration. This is in line with the observations made for *para*-MLL,^37^ for which with increasing Ln concentration, the maximum at 251 nm gradually decreased. Interestingly, the absorbance was further reduced after centrifugation and re-measurement of the supernatant, which led to the assumption that the *para*-MLL–Ln^3+^ complex was removed from the solution, most likely by precipitation.^37^ Therefore, the solutions after the final titration step in the experiments with the MLL derivatives were centrifuged and the supernatant re-measured as well, yielding similar results. Comparing the titrations of La^3+^ and Lu^3+^, it could be cautiously assumed that due to a more pronounced decrease in the absorption of Lu^3+^ the resulting complexes of the later Lns are even less soluble than the ones with earlier Lns. Nevertheless, it should be noted that for the titration of *ortho*-MLL with Lu^3+^ an isosbestic point at 318 nm can be detected, hinting toward a possible shift in absorbance upon formation of a slightly soluble *ortho*-MLL–Lu^3+^-complex. However, as an even further decrease of the absorbance maxima can be observed after centrifugation and re-measurement of the last titration step, the data still indicate that the majority of the formed *ortho*-MLL–Lu^3+^ complex precipitates and we thus cannot comment on the involvement of the hydroxyl group in binding from these data. Similar observations can be made for the titrations with Nd^3+^ and Eu^3+^ (Fig. S3, SI). In conclusion, the UV-vis data strongly suggest that, similar to *para*-MLL, both *meta*-MLL and *ortho*-MLL seem to form poorly soluble complexes with Lns, thereby preventing gaining insights into the coordinating residues using this method. Given the poor solubility of these complexes, NMR investigations are also unsuitable for obtaining a deeper understanding of the Ln binding sites.

### Solubility products *via* time-resolved laser-induced fluorescence spectroscopy

**Fig. 4:**
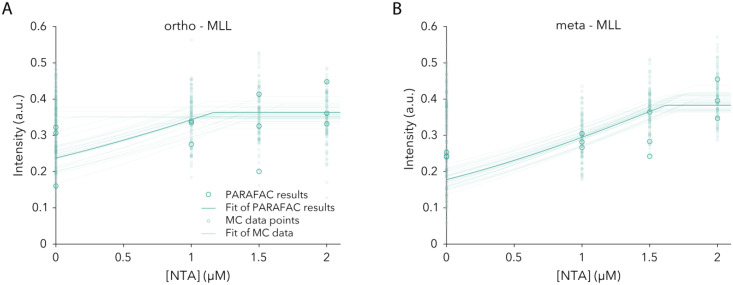
PARAFAC result and fit of the competition experiments between (A) *ortho*-MLL or (B) *meta*-MLL, NTA, and Eu^3+^. Large open circles represent PARAFAC-derived Eu^3+^ luminescence intensities from triplicates of three independent series containing fixed concentrations of *x*-MLL (2 µM) and EuCl_3_ (2 µM). Small transparent circles show Monte Carlo-sampled data points from 100 runs, reflecting the propagated uncertainty of the fit. The bold line represents the best fit to the PARAFAC results; the bundle of transparent lines represents the corresponding fits of the MC data. More detailed experimental parameters and the speciation model used are provided in the SI.

The UV-vis measurements revealed that the chelators form poorly soluble complexes, as demonstrated by precipitation observed during the titration experiments. While this confirmed complex formation, precipitation made it impossible to quantitatively determine the stability constants or solubility products from absorption data alone. A similar challenge was previously encountered with *para*-MLL, where a competition experiment using TRLFS with Eu^3+^ and nitrilotriacetic acid (NTA) was successfully employed for thermodynamic chracterization.^37^ Following this approach, a competition experiment was designed in which Eu^3+^ (2 µM) and the respective *x*-MLL derivative (2 µM) were combined with increasing concentrations of nitrilotriacetic acid (NTA, 0–2 µM) as a competing ligand of known affinity. Based on prior experience, NTA concentrations were limited to 0–2 µM to remain within the concentration range where only the 1 : 1 Eu^3+^–NTA complex is present, to keep the speciation model as simple as possible. After precipitation of the Eu^3+^–*x*-MLL complex and its removal by centrifugation, each sample was subsequently acidified with HCl to dissociate any remaining soluble Eu^3+^ complexes and convert all europium to its aquo ion form. The resulting signal directly reflects the total soluble Eu^3+^ fraction – comprising both free Eu^3+^ and Eu^3+^ previously bound to NTA – whose NTA-dependent trend was modeled using the well-characterized 1 : 1 Eu^3+^–NTA formation constant (log *β* = 11.45, log *K*_a_ = 7.9 at pH 6.0, *I* = 100 mM KCl)^44^ as the only fixed parameter with *K*_SP_ = [*x*-MLL^3−^][Eu^3+^] as the sole unknown term. PARAFAC decomposition of the TRLFS data was performed on three independent triplicates, and the *K*_SP_ value as well as its uncertainty were extracted *via* 100 Monte Carlo runs (Fig. 4).

The experiments yielded solubility products for *ortho*-MLL–Eu^3+^ (log *K*_SP_ = [*ortho*-MLL^−3^] × [Eu^3+^]): −11.72 ± 0.18 mol^2^ L^−2^ and for *meta*-MLL–Eu^3+^ (log *K*_SP_ = [*meta*-MLL^−3^] × [Eu^3+^]): −11.88 ± 0.12 mol^2^ L^−2^ (Fig. 4). The results revealed a trend in the solubility products, with the *para*-MLL–Eu^3+^ complex (log *K*_SP_ = −12.07 ± 0.24 mol^2^ L^−2^)^37^ being the least soluble, followed by *meta*-MLL, while the *ortho*-MLL–Eu^3+^ complex showed the highest solubility product. This indicates that the *para* positioning of the hydroxyl group in natural MLL yields a less soluble and thus potentially thermodynamically more stable Eu^3+^ complex than the *ortho*-derivative, suggesting that this configuration is more favorable for Ln binding.

### Chromeazurol S competition experiment

**Fig. 5:**
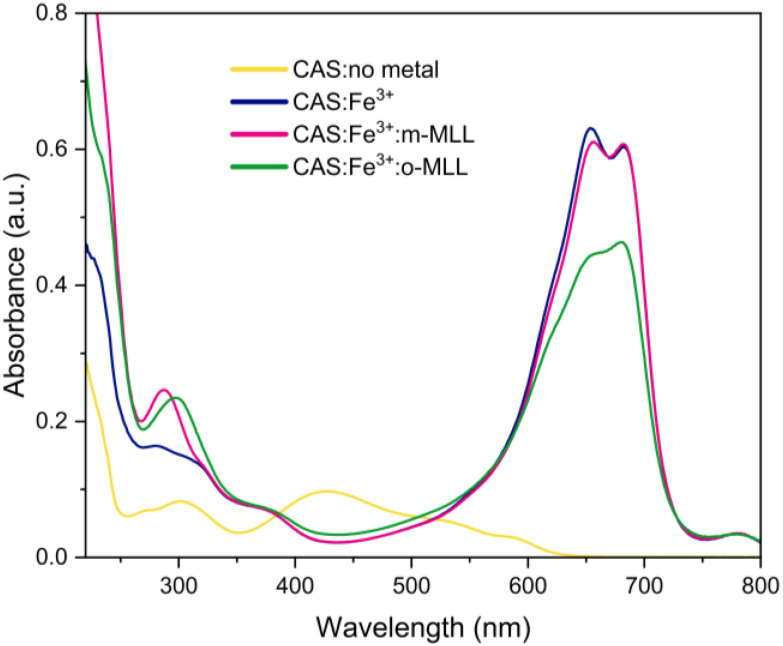
CAS competition experiment of *ortho*-/*meta*-MLL in MOPSO buffer (10 mM, 100 mM KCl, pH 6.0) with FeCl_3_ (final concentration: CAS/HDTMA (18.75 μM/200 μM), FeCl_3_ (18.75 μM), and ligand (18.75 μM)).

As previously stated in the literature,^35,37^ there might be a potential link between Ln^3+^ and Fe^3+^ uptake and their metabolism. Consequently, we were interested in comparing the Fe^3+^ binding properties of these novel chelators to the natural *para*-MLL. To evaluate Fe^3+^ binding of siderophores, Schwyn *et al.*^45^ established the competitive chromeazurol S (CAS)/hexadecyltrimethyl-ammonium bromide (HDTMA) assay which can be easily followed by UV-vis spectroscopy. In this competition experiment a CAS/HDTMA–Fe^3+^ complex competes with another ligand for Fe^3+^. If the added ligand is capable of acquiring Fe^3+^ from the existing CAS/HDTMA complex, a higher Fe^3+^-binding affinity of that ligand compared to that of CAS/HDTMA can be assumed. In the case of the literature-known siderophore RPB B,^32^ the molecule has been reported to be able to remove ferric iron from the CAS/HDTMA–Fe^3+^ complex.^37^ In contrast, only a minor decrease in absorption was observed for *para*-MLL, indicating a smaller affinity for iron than that of the CAS/HDTMA complex.^37^ The experimental data for *ortho*- and *meta*-MLL are shown in Fig. 5. In the case of *meta*-MLL no decrease of absorbance is visible after the addition of the chelator, suggesting, if at all, a lower iron affinity than that of CAS/HDTMA. However, the addition of o*rtho*-MLL resulted in a significant decrease of the CAS/Fe^3+^ maxima at 653 nm, indicating that this molecule is capable of binding iron from the CAS/HDTMA complex.

To further verify the stronger iron binding affinity of *ortho*-MLL, we worked with an excess of a chelator and could show that with higher concentrations of *ortho*-MLL an even further decrease of the CAS/HDTMA/Fe^3+^ maximum is visible (Fig. S4, SI). These results strongly suggest that changing the position of the hydroxyl group to the *ortho*-position does improve the iron affinity of MLL and furthermore indicates that the hydroxyl group is indeed involved in iron-binding in *ortho*-MLL. This could be possible, as a bidentate binding mode with the neighboring carbonyl group of the amide can be formed in *ortho*-MLL in contrast to the natural chelator *para*-MLL, making it a more attractive ligand from a coordination chemistry point of view. A decrease in the CAS/HDTMA/Fe^3+^ maximum would be expected to be accompanied by a corresponding increase in the free CAS absorption at 420 nm, which is not observed. However, this hypothesis is purely preliminary and further thorough investigation is required before the existence of such a complex can be proven. Experiments toward obtaining X-ray crystal structures of all MLL–Ln are still ongoing to confirm the exact coordination environment and determine whether the 4-hydroxy benzoate groups and other functional groups are involved in metal binding.

### Lanthanide binding studies *via* ion mobility mass spectrometry measurements

**Fig. 6:**
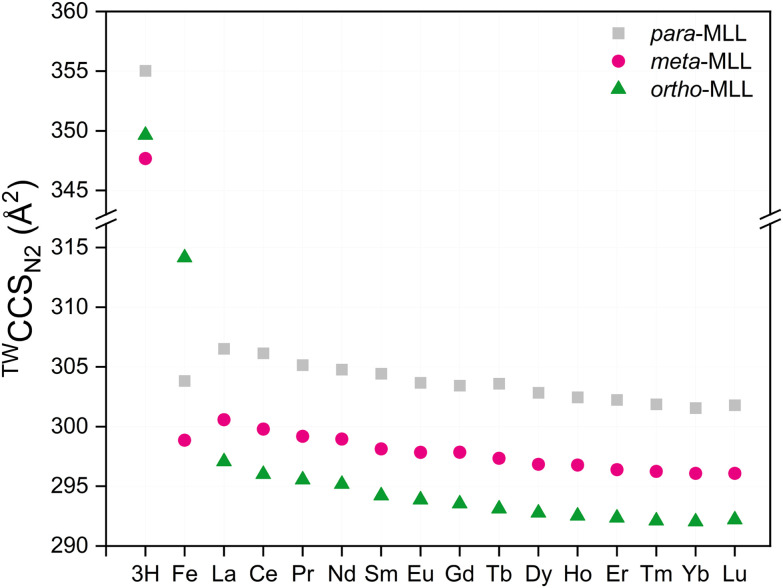
Experimental ^TW^CCS_N2_ of [*para*^37^-/*meta*-/*ortho*-MLL−H+M]^2+^ (M = 3H^+^, Fe^3+^, Ln^3+^ except Pm).

Gaining structural insight into MLL–Ln^3+^ complexes requires an approach that is independent of the limitation incurred by precipitation in solution. A combination of mass spectrometry (MS) and ion mobility spectrometry (IMS), termed IMS-MS, provides such a framework by enabling the characterization of chelator–metal complexes in the gas phase. This approach has been applied successfully across diverse siderophore systems, resolving co-produced structurally similar hydroxamate-based ferrioxamines,^46^ identifying metal-binding molecules in complex bacterial supernatants of marine *Micromonospora* sp.^47^ and discovering new pyoverdine derivatives in *Pseudomonas* samples.^48^ Building on our successful employment of IMS-MS in studying RPB and *para*-MLL,^37^ we again reverted to this method in order to gain further insights into the formed complexes. IMS-MS is sensitive not only to the mass, but also to the shape of isolated ions in the gas phase. In IMS, the ions are transported by an electrical field through a gas-filled drift cell (typically 1–2 mbar of nitrogen) and their arrival time distribution (ATD) is measured. The ATD is a function of the applied field, temperature and pressure, which can then be deconvoluted to ion mobility and finally into a collision cross section (CCS).^49^ These values can then be compared to theoretically calculated ones to confirm or rule out possible isomeric structures. The MLL derivatives were analysed using a Waters Select Series Cyclic IMS by travelling wave (TW) IMS-MS and [*x*-MLL−H+M]^2+^ (M = 3H^+^, Fe^3+^, Ln^3+^ except Pm; *x* = *ortho*, *meta*) complexes were found, which align with the species reported in the literature for *para*-MLL and RPB B.^37^ An overview of the mean ^TW^CCS_N2_ values of the observed doubly charged complexes is shown in Fig. 6. Comparing the CCS values of the [*x*-MLL−H+Ln]^2+^ (*x* = *ortho*, *meta*, *para*) complexes among themselves, the lanthanide contraction is clearly visible for all MLL derivatives, as a steady decrease of the CCS values from the La^3+^ complexes to the Lu^3+^ complex can be observed, which is in line with previous literature.^37,50^ The metal-free doubly protonated species exhibit a larger CCS around 350 Å^2^ as expected for the more flexible form. When comparing the three MLL derivatives with each other, the *para*-MLL complexes possess the largest CCS values followed by the *meta*-MLL complexes, while the complexes obtained for *ortho*-MLL show the smallest CCS values.

**Fig. 7:**
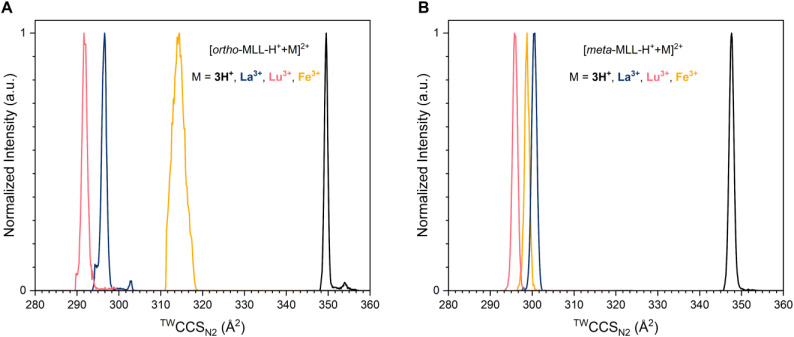
Experimental ^TW^CCS_N2_ of *x*-MLL (A: *x* = *ortho*; B: *x* = *meta*) complexes as [*meta*-/*ortho*-MLL−H+La/Fe]^2+^ and as the protonated ion [*meta*-/*ortho*-MLL+2H]^2+^.

When taking a closer look at Fig. 6, the [*ortho*-MLL−H+Fe]^2+^ complex stands out. The CCS value of 314 Å^2^ strongly deviates from the CCS values obtained for the *para*- and *meta*-MLL iron complexes with values of 304 and 299 Å^2^, respectively. Furthermore, the broad peak of the [*ortho*-MLL−H+Fe]^2+^ complex (Fig. 7) also indicates the existence of multiple isomers. This observation aligns with the data from the CAS experiment, showing a different binding behavior toward ferric iron for this ligand only. This demonstrates the positional impact of the hydroxyl group within the hydroxy benzoate moiety on metal binding.

**Fig. 8:**
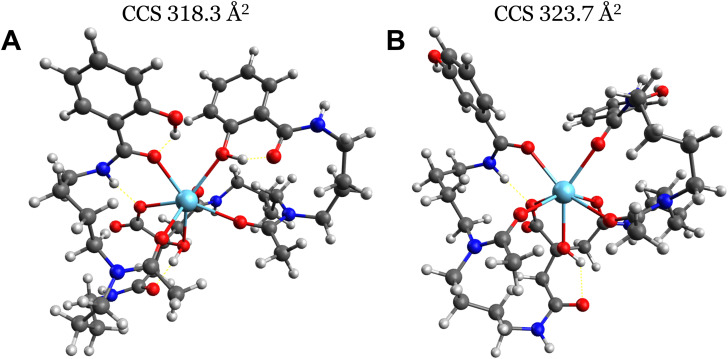
DFT optimized structures of [*x*-MLL−H+La]^2+^ (*x* = *ortho* (A); *meta* (B)) together with calculated CCS.

To gain structural insights into the complexes observed *via* IMS-MS, density functional theory calculations (DFT) based on the ones previously published for *para*-MLL have been conducted for [*meta*-/*ortho*-MLL−H+M]^2+^ (M = 3H^+^, La^3+^, Lu^3+^ and Fe^3+^).^37^ In all cases we find several structures within 1 eV of the lowest energy conformation (Tables S1 and S2). For M = La^3+^, in the lowest energy structure, the metal center is coordinated by seven oxygen atoms, specifically the oxygen atoms from the acetyl, the amide and the citrate groups. An interesting feature is the hydroxyl group positioning of the hydroxy benzoate moiety, which is bent toward the metal center in the *ortho* form while being turned away from the center in the *meta* derivative (Fig. 8). This is consistent with the experimental IMS-MS data, since *ortho*-MLL exhibits the smallest CCS for the lanthanide series, which is reflected by the inward coordination of the hydroxyl group, in contrast to *para*-MLL where the group is bent away from the complex center, therefore showing a larger CCS.^37^

**Fig. 9:**
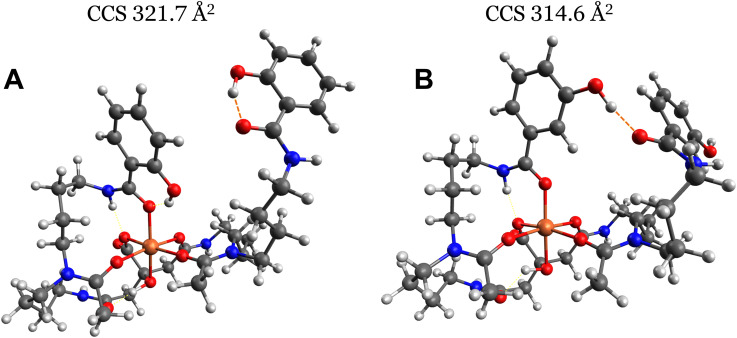
DFT optimized structures of [*x*-MLL−H+Fe]^2+^ (*x* = *ortho* (A); *meta* (B)) together with calculated CCS.

Interestingly, the CCS of the [*ortho*-MLL−H+Fe]^2+^ complex is significantly larger than those of the corresponding Ln complexes, which is surprising given the comparable CCS observed for all [*meta*-MLL−H+M]^2+^ complexes of M = Ln^3+^ and Fe^3+^ (Fig. 6). The respective DFT-optimized lowest energy structures for the *meta* and *ortho* complexes are shown in Fig. 9. In both cases the iron metal center is coordinated by six oxygen atoms and in contrast to the Ln^3+^ complexes, one hydroxy benzoate moiety bends away from the metal center. In the [*meta*-MLL−H+Fe]^2+^ complex a hydrogen bond between one of the amide oxygens and the hydroxyl group of the opposite hydroxy benzoate moiety (Fig. 9B, orange dashed line) stabilizes a compact structure with comparatively small calculated CCS (314.6 Å^2^). In [*ortho*-MLL−H+Fe]^2+^ a hydrogen bond between the amide oxygen and the *ortho*-hydroxyl group of the neighboring hydroxy benzoate moiety is formed (Fig. 9A, orange dashed line). As a consequence, this phenyl group is free to bend away from the metal center and therefore the calculated CCS is significantly larger (321.7 Å^2^). The experimental peak is rather broad (Fig. 7), which is likely due to the fact that in IMS measurements at room temperature several variations (energetically close, but with different CCS) with the hydroxy benzoate moiety pointing in various directions away from the metal center are probed simultaneously.

Note that the calculated CCS of the energetically best structures (Fig. 8, 9 and S7/S8, SI) are based on the trajectory method calculations. Grounded on the method (see the SI for details), their absolute values deviate in small percentages (2%–7%) from the experimental ones. However, they reproduce trends such as the lanthanide contraction from early to late Lns by a decrease of the CCS values along the whole Ln series. Furthermore, the metal-free doubly charged species show larger CCS values, perfectly fitting the experimental data. The calculations generally tend to overestimate the experimental CCS, which is in accordance with previous results.^37^ Nevertheless, it is important to note that these are gas phase investigations of doubly charged [*x*-MLL−H+Ln]^2+^ complexes. In solution, especially for Lns, it is possible that additional ligands such as water are also coordinated and the protonation state of the complexes is different. Overall, the DFT calculations confirm the CCS in the gas phase and show differences between *ortho*-, *meta*- and *para*-MLL.

## Conclusion

Two novel derivatives, *ortho*-MLL and *meta*-MLL, of the natural metallophore *para*-MLL, have been successfully synthesized and their metal binding properties toward trivalent Lns and ferric iron investigated. We showed that both chelators behave similarly regarding their precipitation with trivalent Lns in solution. Nevertheless, the experimental data revealed that the position of the hydroxyl group at the aromatic ring does influence metal binding. TRLFS competition experiments with NTA successfully circumvented the precipitation limitations and enabled the determination of solubility products of Eu^3+^-complexes with the artificial chelators. These results demonstrated that the natural *para*-MLL forms the thermodynamically most stable Eu^3+^-complex, showing the importance of hydroxyl group positioning in Ln binding. Analyzing the iron-binding properties *via* a CAS competition experiment revealed that, in contrast to *para*- and *meta*-MLL, *ortho*-MLL appeared capable of binding to iron from the CAS/HDTMA-Fe^3+^ complex. IMS-MS measurements displayed by comparison of the mean ^TW^CCS_N2_ values of the observed doubly charged Ln complexes [*x*-MLL−H+M]^2+^ (M = 3H^+^, Fe^3+^, Ln^3+^ except Pm; *x* = *ortho*, *meta*, *para*) that all MLL Ln-complexes follow the Ln contraction and show narrow peaks with both ligands. Interestingly, *ortho*-MLL in combination with Fe^3+^ showed a broad peak instead of the narrow peak observed for *meta*-MLL, hinting toward the formation of multiple isomers/conformers in the gas phase. DFT calculations of the Ln complexes presented differences in the spatial coordination of the hydroxyl group, wherein the *meta*-MLL is turned “outward” and the *ortho*-MLL “inward” to the metal center, suggesting a potential involvement in metal binding of this functional group. While gas-phase techniques have provided initial 3D coordination insights, experiments toward obtaining X-ray crystal structures of all MLL–Ln^3+^ complexes are still ongoing to further elucidate the coordination environment of the formed complexes.

## Author contributions

MWM: writing – original draft, visualization, methodology, and investigation (synthesis and metal binding experiments). SLH: investigation (synthesis and metal binding experiments of *meta*-MLL). OLH: investigation (synthesis and metal binding experiments of *ortho*-MLL). PW: writing – original draft, investigation (cIMS measurements and DFT calc. of *meta*- and *ortho*-MLL). BD: investigation (TRLFS measurements of *meta*- and *ortho*-MLL). LJD and SMGT: conceptualization and methodology, supervision, and project administration. LJD and PW: funding acquisition and resources. All authors were involved in writing – review & editing.

## Conflicts of interest

The authors declare no conflicts of interest.

## Supplementary Material

DT-055-D6DT00963H-s001

## Data Availability

Primary data concerning UV–vis (.csv), TRLFS (.sif), NMR (Bruker raw data) and quantum chemical calculations (.xyz) shown in this manuscript and its Supporting Information are available at the RODARE repository: https://doi.org/10.14278/rodare.4748. Further information is also included in the Supporting Information (SI). See DOI: https://doi.org/10.1039/d6dt00963h. The authors have cited additional references within the SI.^51–65^
