# Supplementary material for: Synthesis of two methylolanthanin derivatives and the investigation of their lanthanide and iron binding capabilities
Source: Dalton Trans. 2026 Jul 14;55(30):11274–86. doi: 10.1039/d6dt00963h (PMC13367227; doi:10.1039/d6dt00963h)
Supplement: DT-055-D6DT00963H-s001 [file DT-055-D6DT00963H-s001.pdf]

Supplementary Information (SI) for Dalton Transactions.
This journal is © The Royal Society of Chemistry 2026

Supporting Information

Synthesis of two methylolanthanin derivatives and the investigation of their lanthanide and iron binding capabilities

Michael W. Mertens,[1,2] Sarah L. Hügel,[1] Oliver Hagen,[1] Sophie M. Gutenthaler-Tietze,[1] Patrick Weis,[3] Björn Drobot,[4] Lena J. Daumann[1]

[1] Heinrich-Heine-University Düsseldorf, Chair of Bioinorganic Chemistry, Universitätsstr. 1, 40225 Düsseldorf, Germany, e-mail: Lena.Daumann@hhu.de

[2] Ludwig Maximilian University of Munich, Department of Chemistry, Butenandtstr. 5-13, 81377 Munich, Germany.

[3] Institute of Physical Chemistry, Karlsruhe Institute of Technology, Fritz-Haber-Weg 2, 76131 Karlsruhe, Germany

[4] Institute of Resource Ecology Biogeochemie, Helmholtz-Zentrum Dresden-Rossendorf e.V., Bautzner Landstraße 400, 01328 Dresden, Germany

Table of Content

1. General Working Methods 3

NMR Spectroscopy 4

Preparative HPLC 6

Liquid Chromatography-Mass Spectrometry 6

Cyclic Ion Mobility Spectrometry-Mass Spectrometry 7

UV-vis Spectroscopy 7

Time-Resolved Laser-Induced Fluorescence Spectroscopy 7

Lyophilization 7

2. Organic Synthesis 8

Meta-MLL 14

Ortho-MLL 20

3. Binding Studies 30

Lanthanide to Ligand Titration Experiments 30

Chrome Azurol S (CAS) Fe^{3+} Competition Experiment 31

Nitrilotriacetic acid (NTA) Eu^{3+} Competition Experiment 31

4. Quantum Chemical Calculations 34

5. Data Availability Statement 36

6. References 36

1. General Working Methods

Reagents, dry solvents and deuterated organic solvents were purchased from commercial suppliers (*abcr, Apollo Scientific, BLDpharm, Carlo Erba reagents, Carl Roth, Eurisotop, Fisher chemical, neoFroxx, Grüssing, Sigma-Aldrich and thermoscientific)* and used without further purification. For liquid chromatography-mass spectrometry (LC-MS) analyses, LC-MS-grade solvents and for preparative high-performance liquid chromatography (HPLC) HPLC-grade solvents (both from *VWR*) were used. Deuterated solvents for nuclear magnetic resonance (NMR) spectroscopy were purchased from *Euroisotop*, *Deutero* and *Sigma-Aldrich*. Demineralized water was further purified before usage by an OmniaTap ultrapure water system from *stakpure* to obtain ultra-pure water type 1 (pH 5.6 and 18.2 MΩ/cm at 25 °C) and used within all experiments if not stated otherwise.

All reactions were performed under inert nitrogen atmosphere unless stated otherwise. Chemicals sensitive to air and/or water were handled using dried solvents and Schlenk techniques. Reactions were carried out in one-neck round-bottomed flasks sealed by a septum, applying inert gas atmosphere (N_2) *via* steel cannulas (0.80 x 40 mm). Silica-coated aluminum plates impregnated with a fluorescence indicator at 254 nm (0.25 mm, 60 Å pore-size, *Merck KGaA*) were used for thin layer chromatography (TLC). Aromatic compounds were detected using ultraviolet (UV) light at λ = 254 nm and 365 nm, if not stated otherwise. TLC plates were bought as 20 × 20 cm sheets, cut by hand and performed on plates with a length (direction of flow) of 6 cm. Substances were applied at a distance of 1 cm to the bottom and the chromatography was stopped when the solvent front was approximately 0.5 – 1 cm from the top edge, resulting in a travel distance of approx. 4 – 4.5 cm for the mobile phase. Flash column chromatography was performed with an *Interchim* puriFlash XS 520+ system using self-packed silica columns (Silica 60, 0.04 – 0.062 mm from *Macherey-Nagel*) for separation. The eluent ratios for TLC and column chromatography are given in percentages by volume. The eluent AM consisted of a 1:9 mixture of an aqueous solution of ammonia (25 %) and methanol. Solvents were removed with a rotary evaporator under reduced pressure at 40 °C unless stated otherwise.

NMR Spectroscopy

^1H and ^{13}C NMR spectra were recorded at the Center for Molecular and Structural Analytics (CeMSA) of the HHU Düsseldorf using an Avance III – 600 system operating at 600 MHz for ^1H- and 151 MHz for $^{13}C\{^1H\}$-spectra at 25 °C. ^1H and ^{13}C chemical shifts are reported in ppm units relative to $CDCl_3$ (δ_H = 7.26 ppm, δ_C = 77.16 ppm (central line of the triplet) or TMSP-d_4 in D_2O (δ_H = 0.00 ppm, δ_C = 0 ppm). Coupling constants (J) are given in Hertz (Hz). The generated data was processed using MestReNova 14.0.1-23559. For signal assignments, two-dimensional correlation spectroscopy (^1H-^1H COSY), heteronuclear single quantum coherence spectroscopy (^1H-^{13}C HSQC) and heterobinuclear multiple bond connectivity spectroscopy (^1H-^{13}C HMBC) were conducted. ^1H- and ^{13}C- signals were assigned to the according H and C atoms of the compounds by arbitrary numbering starting from 1, if such assignment was possible. Signal multiplicities were characterized as singlet (s), doublet (d), triplet (t), quartet (q), multiplett (m) and combinations thereof.

Quantitative NMR analysis (*q*NMR) was performed on a Bruker Avance NEO Evo – 600 operated at 600 MHz by the CeMSA of the HHU. The following acquisition settings were used for *meta*-MLL: 60 s delay, 5 acquisition time, 128 scans; and for *ortho*-MLL: 60 s delay, 1 s acquisition time, 32 scans. The procedure for *ortho*-MLL was performed according to a procedure described in the literature.[1] Due to overlapping proton signals in the NMR spectrum a different procedure for the *meta*-MLL quantification was used. A 0.5 mg/mL stock solution of the certified reference material calcium formate in D_2O + 0.03 % TMSP-d_4 was prepared. A defined amount of *meta*-MLL stock solution in water was lyophilized and subsequently re-dissolved in D_2O. The calcium formate solution was then diluted 1:10 with the *meta*-MLL-D_2O-solution. To calculate the concentration of the x-MLL (x = *ortho*, *meta*) stock in water, the following equation 1 was used with I standing for integral, N for number of protons and P for purity according to a literature procedure.[1] The corresponding *q*NMR spectra are shown in Figure S1 and S2.

$$(1) \qquad c_x = \frac{I_x}{I_{Std}} \times \frac{N_{Std}}{N_x} \times c_{Std} \times P_{Std}$$

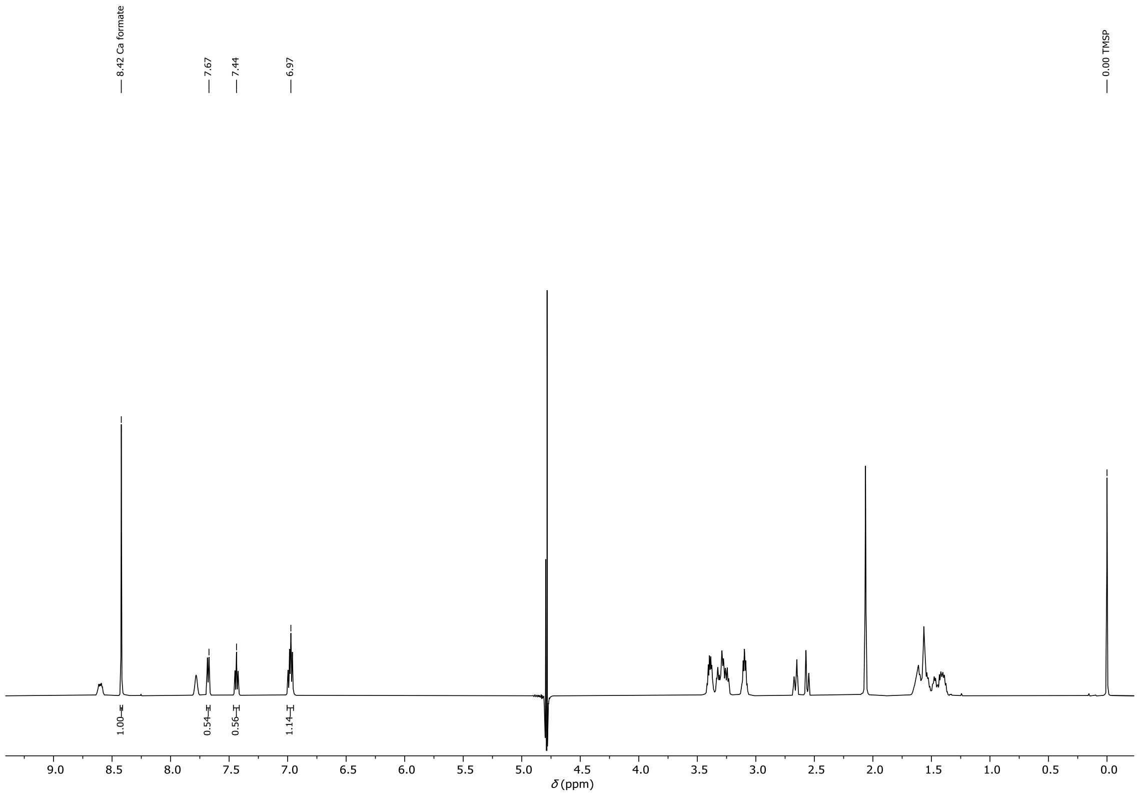

Figure S1: q*NMR (9:1 H_2O:D_2O+0.003 % TMSP*-d*4, 600 MHz) of* ortho-*MLL with the certified reference material calcium formate. The individual integrals of the aromatic signals were used for the quantification.*

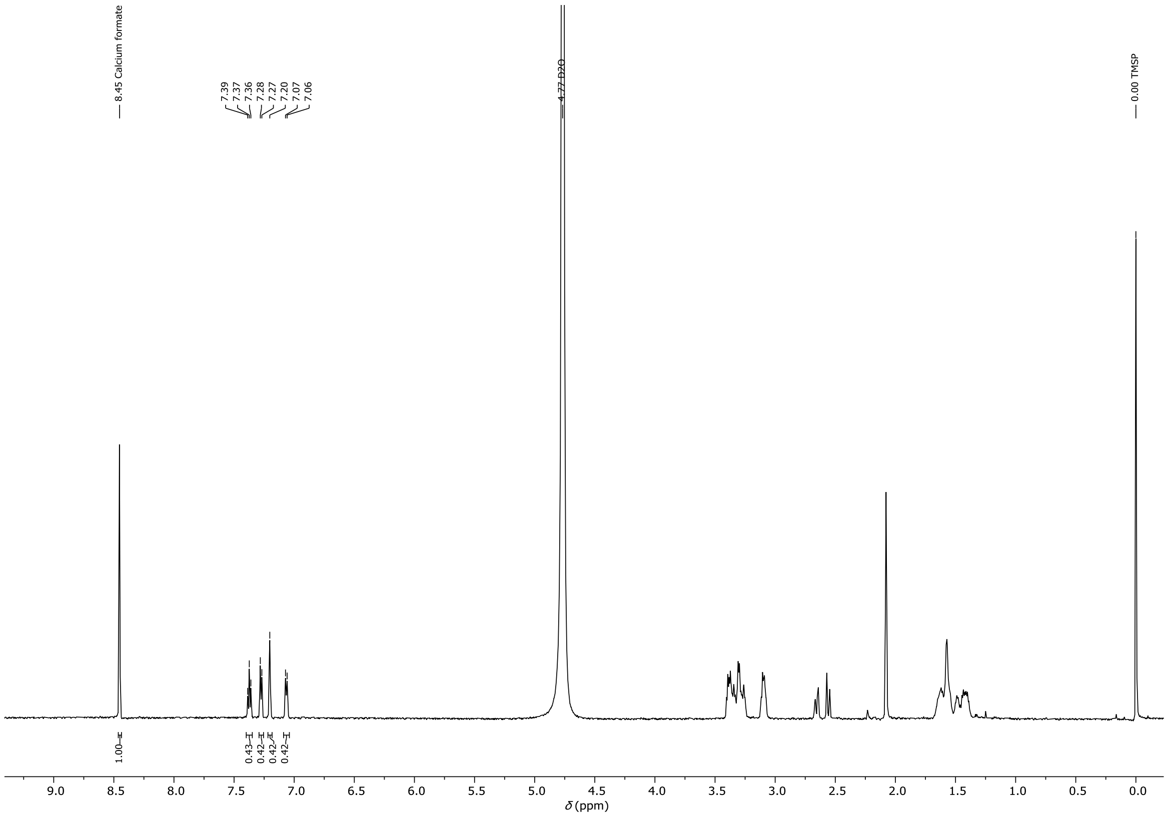

Figure S2: q*NMR (D_2O+0.003 % TMSP*-d*4, 600 MHz) of* meta-*MLL with the certified reference material calcium formate. The individual integrals of the aromatic signals were used for the quantification.*

Preparative HPLC

Preparative HPLC was performed on a preparative *Agilent* Prep HPLC Infinity II 1260 equipped with a G7161A preparative binary pump, a G7165A multiple wavelength detector (MWD), a G1364E fraction collector and a manual injector. Purifications were performed for *meta*-MLL with a *Dr. Maisch* ReproSil-Pur 120 C18-AQ semi-preparative column (250 mm x 10 mm, 5 µm) and for *ortho*-MLL with a *Dr. Maisch* ReproSil Gold 120 C18 column (250 mm x 20 mm, 5 µm) using varying gradients of an acetonitrile/water mixture containing 0.1 % formic acid (FA) and varying flow rates.

Liquid Chromatography-Mass Spectrometry

Low-resolution electrospray mass spectra, which were used for both reaction monitoring and HPLC method development, were recorded on an *Agilent* MSD XT equipped with a 1260 Infinity II HPLC in alternate electrospray ionisation (ESI) mode. The HPLC-system was equipped with a G7115A Diode Array Detector (DAD), a G7116A column thermostat, a G7167A multisampler and a G7104C flexible pump. To assess the reaction's progress, the sample was implemented *via* direct injection with a 1:1 acetonitrile/water mixture containing 0.1 % FA each and using a flow rate of 0.7 mL/min. Reaction control was also performed by implementing the samples using reversed-phase C18 columns and separated by varying flow rates and gradients of an acetonitrile/water mixture containing 0.1 % FA. For separation the following columns were used: *Agilent* Zorbax SB-C18, 50 x 2.1 mm, *Dr. Maisch* ReproSil-Pur 120 C18-AQ, 250 x 4.6 mm, 5 µm and a *Dr. Maisch* ReproSil Gold 120 C18. 250 x 4.6 mm, 5 µm. The latter two columns were used for method development for the purification *via* preparative HPLC. Detection was carried out from *m/z* 80 to 1500 in positive ion mode with a source voltage of 1500 V and the gas temperature was kept at 250 °C. Samples were diluted properly using either solely methanol or a 1:1 acetonitrile/water mixture and syringe filtered (0.2 µm, PTFE) prior to injection. (ESI)-high-resolution mass spectrometry (HRMS) was performed at CeMSA at HHU Düsseldorf on a *Bruker* Daltonics UHR-QTOF maXis 4G system and a UHPLC-system Ultimate 3000 RS from Dionex in positive ion mode.

Cyclic Ion Mobility Spectrometry-Mass Spectrometry

Samples were prepared by mixing an aqueous solution of the respective MLL (1 µM, 30 µL) with an aqueous solution of MCl_3 (100 µM, 30 µL; M = Fe^{3+}, Ln^{3+} except Pm) which was then further diluted with 100 µL MeOH. Arrival time distributions (ATD) were measured with a *Waters* Select Series Cyclic instrument. The instrument comprises an electrospray ion source, a quadrupole mass filter, a cIMS cell, and a high-resolution time-of-flight mass spectrometer. In the IMS cell the ion packet is guided by an oscillating electrical field („travelling wave“) through 1.7 mbar of nitrogen as drift gas. The wave parameters used for $[x\text{-MLL}-H+M]^{2+}$ (x = *ortho*, *meta*, *para*) were: wave height 22 V and wave speed 375 m/s. Up to 20 cycles were measured. The measured ATDs times were transferred into $^{TW}CCS_{N2}$, by a calibration procedure[2,3] with a set of calibrant ions („agilent tune mix“ (*Agilent*, G1969-8500)) with known CCS.[4] With this procedure we obtain highly reproducible $^{TW}CCS_{N2}$ with statistical errors well below 0.5 %.

UV-vis Spectroscopy

UV-vis measurements were carried out on an *Agilent* Cary 60 UV-vis instrument operated by Cary WinUV Scan Application Version 5.3.0.1079 software and equipped with a Peltier element. Spectra were recorded from 900 or 800 nm to 200 nm with a scan rate of 600 nm/min at 25 °C. Samples were either measured in *Brand* UV micro cuvettes or a *Hellma* QS Ultra-Micro cell, both with 1 cm pathlength. All spectra were blank corrected by subtracting a spectrum of the used solvent or buffer. The obtained data were analysed and plotted using OriginPro 2025 (64-bit) 10.2.0.188 (Academic).

Time-Resolved Laser-Induced Fluorescence Spectroscopy

Time-resolved laser-induced fluorescence spectroscopy (TRLFS) measurements were performed with the following setup: Laser (*Ekspla*, NT 230, ~5 ns pulse); spectrograph (*Andor Technology*, SR-303i-A) and ICCD camera (*Andor Technology*, i Star DH320T-18U-63). For the Eu^{3+} experiments the excitation wavelength was set to 394 nm.

Lyophilization

For lyophilization an Alpha 3-4 LSC basic from *Martin Christ* with a condenser temperature of –105 °C was used. If light sensitive compounds had to be freeze-dried, the vessel was wrapped in aluminium foil prior to lyophilization to avoid decomposition.

2. Organic Synthesis

Compound 1

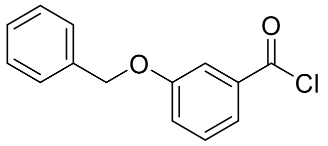

1

The reaction was performed according to a modified literature procedure.[3] A flame-dried flask was charged with 3-benzyloxybenzoic acid (107 mg, 469 µmol, 1.00 equiv.) and dissolved in 3 mL of absolute dichloromethane and 1.2 mL of absolute toluene before oxalyl chloride (100 µL, 1.17 mmol, 2.49 equiv.) was added. Subsequently, three drops of *N,N*-dimethylformamide (DMF) were added every 30 min for 1.6 hours using a syringe until no further gas formation was observed. Meanwhile the reaction mixture turned from a colorless solution to a yellow suspension. The reaction was stopped by removing the volatiles under reduced pressure yielding the crude product as pale-yellow solid, which was used without further purification immediately for the following reaction. When dissolving compound **1** for the following reaction in dichloromethane, it was observed that not only the desired product was formed, but also a white solid which remains insoluble, indicating the formation of a byproduct.

Compound 2

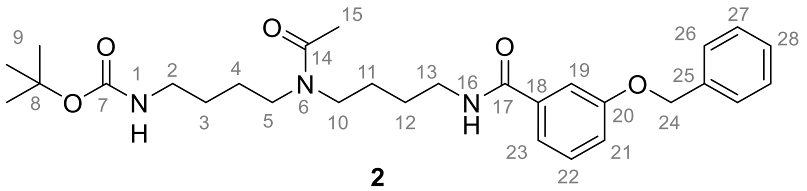

2

The reaction was performed according to a modified literature procedure.[3] A flame-dried flask was charged with *tert*-butyl-(4-(*N*-(4-aminobutyl)acetamido)butyl)carbamate (107 mg, 355 µmol, 1.00 equiv.), which was synthesized prior according to a literature procedure,[3] and dissolved in 6 mL of absolute dichloromethane. Subsequently, triethylamine (0.13 mL, 934 µmol, 2.64 equiv.) was added before the reaction mixture was cooled to 0 °C. Meanwhile, compound **1** (123 mg, 499 µmol, 1.40 equiv.) was dissolved in 6 mL of absolute dichloromethane under inert nitrogen atmosphere and transferred dropwise to the reaction solution using a syringe. The reaction mixture changed its color

from pale yellow over dark yellow to a deep brown solution during the addition of compound **1**. After one hour, the reaction mixture was left to warm to 25 °C. Formation of a brown solid was observed. The reaction was stopped by removing the volatiles under reduced pressure after TLC (10% AM, 90% DCM) indicated full consumption of the starting material yielding the crude product as a mixture of brown solid and brown oil. Purification of the crude was performed by column chromatography (silica; grading from 2 % AM, 98 % DCM to 4 % AM, 96 % DCM to 15 % AM, 85 % DCM) to obtain product **2** (68.1 mg, 133 µmol, 38 %) as pale-yellow oil.

R_f = 0.48 (UV; 10 % AM, 90 % DCM).

^{1}H NMR (600 MHz, $CDCl_3$, 295 K): δ = 7.53 (t, J = 2.1 Hz, 1H, 19-H), 7.46 - 7.36 (m, 5H, 26-H, 21-H, 22-H, 23-H), 7.33 (dt, J = 10.6 Hz, 6.4 Hz, 2H, 27-H), 7.09 (dd, J = 8.2, 2.8 Hz, 1H, 28-H), 6.91 (s, 0.5H, N16H), 6.36 (s, 0.5H, N16H), 5.11 (d, J = 6.5 Hz, 2H, 24-H), 4.65 (d, J = 39.6 Hz, 1H, N1H), 3.50 (dq, J = 16.0, 5.8 Hz, 2H, 13-H), 3.37 - 3.07 (m, 6H, 2-H, 5-H, 10-H), 2.07 (d, J = 3.3 Hz, 3H, 15-H), 1.68 - 1.46 (m, 10H, 3-H, 4-H, 11-H, 12-H), 1.43 (d, J = 4.2 Hz, 9H, 9-H) ppm.

^{13}C NMR (151 MHz, $CDCl_3$, 295 K): δ = 170.3 (C14), 167.4 (C17), 159.1 (C20), 136.8 (C18/C25), 136.2 (C18/C25), 129.7 (C22), 128.8 (C21), 128.2 (C23), 127.7 (C26), 119.0 (C27), 118.5 (C28), 113.3 (C19), 70.3 (C24), 48.7 (C5/C10), 45.1 (C5/C10), 39.6 (C13), 28.6 (C9), 27.7 (C3/C4/C11/C12), 26.2 (C3/C4/C11/C12), 25.4 (C3/C4/C11/C12), 21.7 (C15) ppm.

HRMS (ESI+) calculated for $[C_{29}H_{11}N_3O_5 + H]^+$ *m/z* 512.3119, found *m/z* 512.3116 with a deviation of −0.5856 ppm.

Compound 3

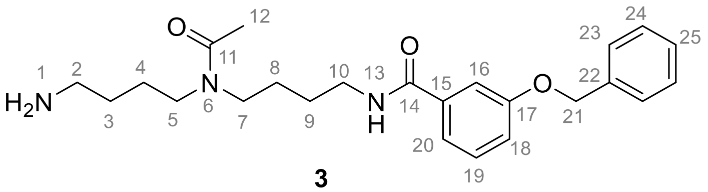

3

The reaction was performed according to a modified literature procedure.[3] Starting material **2** (143 mg, 279 µmol, 1.00 equiv.) was dissolved in 7 mL of dichloromethane and cooled to 0 °C under standard atmospheric conditions. Subsequently, concentrated trifluoroacetic acid (1.07 mL, 14.0 mmol, 50.2 equiv.) was dissolved in 2 mL dichloromethane before its dropwise addition to the reaction mixture. The cooling bath was removed after 15 min and the solution was left to stir at 25 °C for another 3 h, at which point TLC and LRMS indicated full consumption of the starting material. The reaction was stopped by removing the volatiles under reduced pressure yielding the crude as yellow oil. The crude product was purified by flash column chromatography (silica; grading from 4 % AM, 96 % DCM over 10 % AM, 90 % DCM and 12 % AM, 88 % DCM to 15 % AM, 85 % DCM) obtaining product **3** as pale-yellow oil (109 mg, 265 µmol, 95 %).

R_f = 0.14 (UV; 10 % AM, 90 % DCM).

^{1}H NMR (600 MHz, $CDCl_3$, 295 K): δ = 7.53 (dd, J = 2.6, 1.6 Hz, 1H, 16-H), 7.46 - 7.42 (m, 2H, 23-H), 7.42 - 7.37 (m, 3H, 24-H, 25-H), 7.36 - 7.28 (m, 2H, 18-H, 19-H), 7.10 (dddd, J = 11.8 Hz, 8.2 Hz, 2.7 Hz, 1.0 Hz, 1H, 20-H), 6.85 (m, 0.5H, N13H), 6.20 (m, 0.5H, N13H), 5.12 (d, J = 6.0 Hz, 2H, 21-H), 3.54 - 3.47 (m, 2H, 10-H), 3.39 - 3.23 (m, 4H, 5-H, 7-H), 2.71 (dt, J = 16.4, 7.0 Hz, 2H, 2-H), 2.08 (d, J = 7.4 Hz, 3H, 12-H), 1.69 - 1.39 (m, 8H, 3-H, 4-H, 8-H, 9-H) ppm.

^{13}C NMR (151 MHz, $CDCl_3$, 295 K): δ = 170.6 (C12), 159.1 (C17), 129.7 (C18/C19), 128.8 (C24/C25), 128.2 (C18/C19), 127.7 (C23), 119.4 (C24/C25), 118.6 (C20), 113.3 (C16), 70.3 (C21), 51.0 (C10), 49.1 (C5/C7), 45.2 (C5/C7), 42.0 (C2), 39.7 (C10), 31.0 (C3/C4/C8/C9), 26.6 (C3/C4/C8/C9), 26.2 (C3/C4/C8/C9), 25.5 (C3/C4/C8/C9), 21.7 (C12) ppm.

HRMS (ESI+) calculated for $[C_{24}H_{33}N_3O_3 + H]^+$ *m/z* 412.2595, found *m/z* 412.2594 with a deviation of −0.170 ppm.

Compound 4

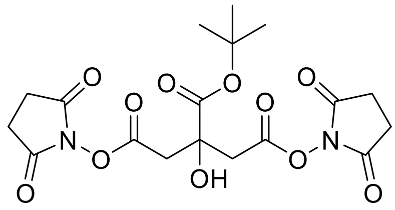

4

The reaction was performed according to a modified literature procedure.[3] Citric acid *tert*-butyl ester (31.3 mg, 126 µmol, 1.00 equiv.), which was synthesized according to a literature procedure[3] prior to this reaction, *N,N'*–dicyclohexylcarbodiimide (DCC, 77,9 mg, 378 µmol, 2.99 equiv.) and *N*–hydroxysuccinimide (NHS, 43.4 mg, 377 µmol, 2.99 equiv.) were dried in three separate flame–dried round bottom flasks under reduced pressure. Subsequently, citric acid *tert*-butyl ester was dissolved in 2 mL of absolute tetrahydrofuran, while DCC and NHS were dissolved in 1 mL of absolute tetrahydrofuran each under inert nitrogen atmosphere. First the NHS solution was added dropwise to the protected citric acid, followed by dropwise addition of the dissolved DCC. The reaction mixture was left to stir at room temperature for 3 h. After 30 min the reaction mixture turned turbid white. The reaction was stopped by removing the solvent under reduced pressure for 1 h yielding the title compound **4** as white solid which was immediately used in the following reaction without further purification.

Benzyl protected *meta*-MLL (5)

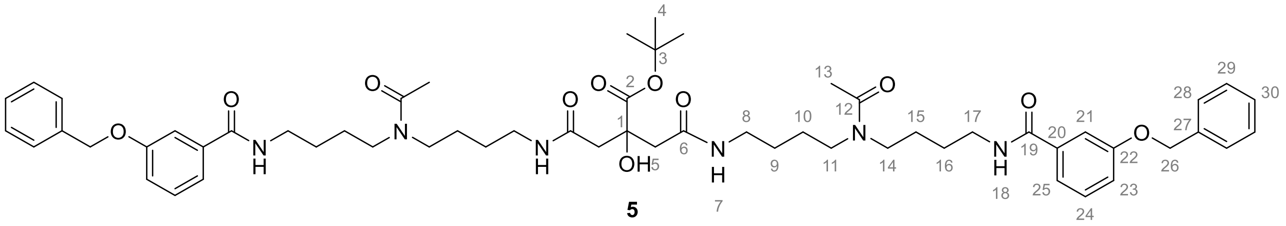

5

The reaction was performed according to a modified literature procedure.[3] A round-bottomed flask was charged with the compound **4** (57.7 mg, 130 µmol, 1.00 equiv.) before 5 mL absolute 1,4-dioxane was added under inert nitrogen atmosphere. Subsequently, the white suspension was cooled to 10 °C. Meanwhile, compound **3** (113 mg, 275 µmol, 2.11 equiv.) was dissolved in 5 mL absolute dichloromethane under inert nitrogen atmosphere before triethylamine (0.27 mL, 1.95 mmol, 14.9 equiv.) was added and left to stir for 25 min at 25 °C. Afterwards, the reaction mixture containing compound **3** and triethylamine was added dropwise to the suspension of compound **4** under exclusion of light. The reaction mixture was left to warm to 25 °C and stirred overnight. It was observed that the reaction mixture changed its color from white to yellow after 1 h. Reaction control *via* TLC and LRMS indicated full consumption of the starting material after 22.2 h. Therefore, the reaction was stopped by removing the volatiles under reduced pressure. The residue was redissolved in 30 mL dichloromethane and washed with 3 x 30 mL of a saturated sodium carbonate solution. The combined aqueous layers were extracted with 2 x 20 mL dichloromethane since TLC still indicated presence of the crude product. The organic layers were combined and dried over anhydrous sodium sulfate before the solvent was removed under reduced pressure yielding the crude product as a mix of yellow oil and solid. The crude product was purified by flash column chromatography (silica; grading from 2 % AM, 98 % DCM over 10 % AM, 90 % DCM to 15 % AM, 85 % DCM). The title compound **5** (83.0 mg, 80.2 µmol, 61 %) was obtained as white crystalline solid after removal of the solvent under reduced pressure and additional lyophilization.

$\mathbf{R_f}$ = 0.36 (UV; 10 % AM, 90 % DCM).

^{1}H NMR (600 MHz, $CDCl_3$, 295 K): δ = 7.50 (ddd, J = 9.2, 2.6, 1.6 Hz, 1H, 21-H), 7.46 (dd, J = 2.8 Hz, 1.4 Hz, 1H, 21-H), 7.44 - 7.40 (m, 4H, 28-H), 7.38 (td, J = 7.5 Hz, 1.6 Hz, 5H, 24-H/25-H/29-H/30-H), 7.35 - 7.29 (m, 5H, 24-H/25-H/29-H/30-H), 7.09 (tdd, J = 8.1 Hz, 2.7 Hz, 1.3 Hz, 2H, 23-H), 6.72 (t, J = 6.0 Hz, 1H, N18H), 5.11 - 5.08 (m, 4H, 26-H), 3.50 - 3.44 (m, 4H, 17-H), 3.37 - 3.12 (m, 12H, 8-H, 11-H, 14-H), 2.74 - 2.52 (m, 4H, 5-H),

2.05 (d, J = 3.5 Hz, 6H, 13-H), 1.66 - 1.46 (m, 16H, 9-H, 10-H, 15-H, 16-H), 1.45 - 1.43 (m, 9H, 4-H) ppm.

^{13}C NMR (151 MHz, $CDCl_3$, 295 K): δ = 173.0 (C2), 170.6 (C12), 167.6 (C19), 159.1 (C22), 136.5 (C27), 129.8 (C24/C25/C29/C30), 128.8 (C24/C25/C29/C30), 128.3 (C24/C25/C29/C30), 127.7 (C28), 119.5 (C24/C25/C29/C30), 119.2 (C24/C25/C29/C30), 118.5 (C23), 113.7 (C21), 113.4 (C21), 82.9 (C3), 74.1 (C1), 70.3 (C26), 49.3 – 48.2 (C8/C11/C14), 45.5 (C8/C11/C14), 39.5 (C17), 27.9 (C4), 27.2 (C9/C10/C15/C16), 27.0 (C9/C10/C15/C16), 26.3 (C9/C10/C15/C16), 25.2 (C9/C10/C15/C16), 21.7 (C13) ppm.

HRMS (ESI+) calculated for $[C_{58}H_{79}N_6O_{11} + H]^+$ m/z 1035.5801, found m/z 1035.5795 with a deviation of −0.5794 ppm.

***Meta*-MLL**

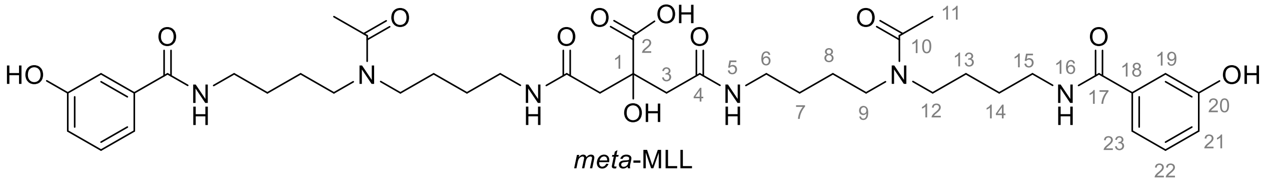

meta-MLL

The reaction was performed according to a modified literature procedure.[3] A round-bottomed flask was charged with compound **5** (70.1 mg, 67.7 µmol, 1.00 equiv.) and dissolved in 3.76 mL concentrated hydrochloric acid under the exclusion of light. Subsequently, 9.4 mL of concentrated hydrochloric acid were added dropwise to the reaction mixture and left to stir for 2 h. LRMS indicated full consumption of the starting material. Therefore, the volatiles were removed under reduced pressure at 35 °C followed by co-evaporation with 2 x 10 mL dichloromethane and drying under reduced pressure over night to remove residual acid. The crude product was obtained as light yellow solid which was redissolved in 10 % ACN in H_2O and purified by preparative HPLC (*Dr. Maisch* ReproSil-Pur 120 C18-AQ semi-preparative column (250 mm x 10 mm, 5 µm), Eluent A: H_2O + 0.1 % FA, Eluent B: ACN + 0.1 % FA, gradient from 2 % B to 25 % B in 5 min, hold for 17 min to 98 % B, flow rate: 4.0 mL/min) yielding ***meta*-MLL** (22.0 mg, 27.4 µmol, 41 %) as colorless solid.

HPLC: $\mathbf{R_t}$ = 18.2 min (254 nm).

^{1}H NMR (600 MHz, D_2O + 0.003 % TMSP-d_4, 295 K): δ = 7.35 (t, J = 7.9 Hz, 2H, 22-H), 7.26 (dt, J = 7.9 Hz, 1.2 Hz, 2H, 23-H), 7.19 (t, J = 2.2 Hz, 2H, 19-H), 7.05 (dddt, J = 8.1 Hz, 2.8 Hz, 1.8 Hz, 0.9 Hz, 2H, 21-H), 3.40 - 3.22 (m, 12H, 9-H, 12-H, 15-H), 3.09 (dt, J = 11.5 Hz, 6.7 Hz, 4H, 6-H), 2.71 - 2.56 (m, 4H, 3-H), 2.06 (d, J = 2.5 Hz, 6H, 11-H), 1.67 - 1.35 (m, 16H, 7-H, 8-H, 13-H, 14-H) ppm.

^{13}C NMR (151 MHz, D_2O + 0.003 % TMSP-d_4, 295 K): δ = 176.6 (C10), 174.7 (C4), 173.2 (C17), 158.8 (C20), 133.3 (C22), 122.6 – 121.5 (m, C23, C21), 116.8 (C19), 77.6 (C1), 51.8 (C9/C12/C15), 48.7 (C9/C12/C15), 47.3 (C3,), 42.4 (C9/C12/C15) 41.8 (C6), 28.7 (C7/C8/C13/C14), 28.6 (C7/C8/C13/C14), 28.1 (C7/C8/C13/C14), 27.2 (C7/C8/C13/C14), 23.5 (C11) ppm.

HRMS (ESI+) calculated for $[C_{40}H_{58}N_6O_{11} + H]^+$ m/z 799.4236, found m/z 799.4235 with a deviation of −0.125 ppm.

Compound 6

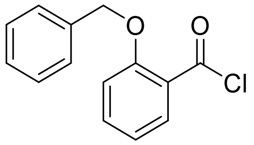

6

The reaction was performed according to a modified literature procedure.[3] In a 100 mL round-bottomed flask 2-(benzyloxy)benzoic acid (276 mg, 1.19 mmol, 1.00 equiv.) was dissolved in 20 mL of absolute dichloromethane and 12 mL of absolute toluene. Then, oxalyl chloride (0.20 mL, 2.29 mmol, 1.92 equiv.) was added dropwise using a syringe. In 30 min intervals, three drops of absolute *N*,*N*-dimethylformamide were added over 1.5 h. The volatiles were removed under reduced pressure to obtain compound **6** as yellow solid, which was then used without further purification in the synthesis of compound **7**.

Compound 7

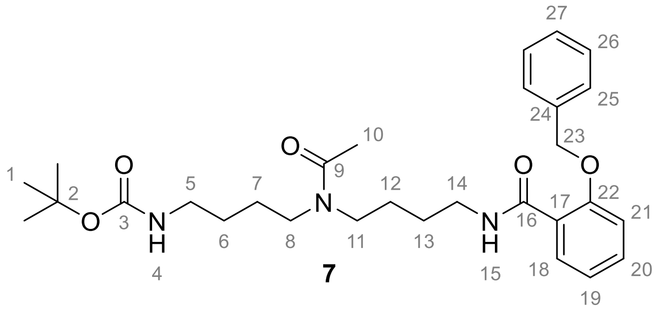

7

The reaction was performed according to a modified literature procedure.[3] The starting material, which was synthesized prior according to a literature procedure,[3] *tert*-butyl-(4-(*N*-(4-aminobutyl)acetamido)butyl)carbamate (325 mg, 1.08 mmol, 1.00 equiv.) was dissolved in a 100 mL round-bottomed flask in 15 mL of absolute dichloromethane. Then triethylamine was added (0.35 mL, 2.51 mmol, 2.33 equiv.) dropwise with a syringe. The reaction was cooled with an ice bath for 1 h. Then compound **6** was dissolved in 20 mL of absolute dichloromethane and transferred dropwise to the starting material with a syringe. The ice bath was removed and after stirring for 17 h, the solution turned orange and LC-MS showed complete conversion of the starting material. The organic reaction mixture was washed with 30 mL of a saturated sodium hydrogen carbonate solution and 30 mL of a saturated sodium chloride solution. The aqueous phase was extracted with 3 x 30 mL dichloromethane, dried over anhydrous sodium sulfate and the volatiles were removed under reduced pressure. The crude product was purified by flash column chromatography

(silica; grading from 2 % AM, 98 % DCM to 10 % AM, 90 % DCM) obtaining compound **7** as colorless oil (372 mg, 727 µmol, 67 %).

R_f = 0.25 (UV; 5 % AM, 95 % DCM).

^{1}H NMR (600 MHz, $CDCl_3$, 295 K): δ = 8.26–8.20 (m, 1H, 18-H), 7.96–7.91 (m, 1H, 15-H), 7.49–7.38 (m, 6H, 20-H, 25-H, 26-H, 27-H), 7.14–7.04 (m, 2H, 19-H, 21-H), 5.18 (d, J = 4.3 Hz, 2H, 23-H), 4.69–4.63 (m, 1H, 4-H), 3.40–3.33 (m, 2H, 14-H), 3.24 (t, J = 7.5 Hz, 2H, 11-H), 3.21–3.10 (m, 2H, 8-H), 3.12-3.08 (m, 2H, 5-H), 2.03 (d, J = 24.5 Hz, 3H, 10-H), 1.58–1.34 (m, 3H, 6-H, 7-H, 12-H, 13H), 1.44 (d, J = 2.9 Hz, 11H, 1-H), 1.40–1.33 (m, 2H, 13-H) ppm.

^{13}C NMR (151 MHz, $CDCl_3$, 295 K) δ = 170.2 (C-9), 165.3 (C-16), 156.9 (C 22), 156.17(C-3), 135.7 (C-18), 132.9 (C-20/C-27), 132.5 (C-20/C-27), 129.1 (C-25/C-26), 128.2 (C-25/C-26), 121.9 (C-19/C-21), 112.7 C-19/C-21), 79.4 (C-2), 71.5 (C-23), 48.4 (C-11), 45.2 (C-8), 39.3 (C-5), 39.0 (C-14), 28.5 (1-C), 27.5 (C-6/C-7/C-12/C-13), 27.0 (C-6/C-7/C-12/C-13), 26.7 (C-6/C-7/C-12/C-13), 26.1 (C-6/C-7/C-12/C-13), 25.1 (C-6/C-7/C-12/C-13), 21.6 (C-10) ppm.

HRMS (ESI+) calculated for $[C_{29}H_{41}N_3O_5 + H]^+$ *m/z* 512.3119, found *m/z* 512.3122 with a deviation of 0.586 ppm.

Compound 8

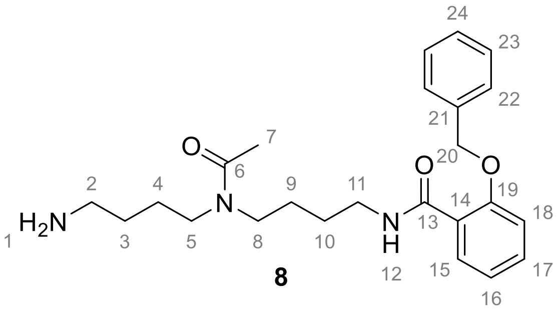

8

The reaction was performed according to a modified literature procedure.[3] In a 100 mL round-bottomed flask, compound **7** (342 mg, 667 µmol, 1.00 equiv.) was dissolved in 8 mL of dichloromethane and cooled with an ice bath. After 15 min, trifluoroacetic acid (1.00 mL, 13.0 mmol, 19.5 equiv.) dissolved in 1 mL of dichloromethane was added with a syringe and cannula and the cooling was removed. After 80 min LC-MS indicated total conversion of the starting material and the volatiles were removed under reduced pressure. The crude oil was purified by flash column chromatography (silica; grading from 5 % AM, 95 % DCM to 10 % AM, 90 % DCM) obtaining compound **8** (253 mg, 616 µmol, 92 %) as colorless oil.

R_f = 0.11 (UV; 10 % AM, 90 % DCM).

^{1}H NMR (600 MHz, $CDCl_3$, 295 K): δ = 8.26–8.20 (m, 1H, 15-H), 7.97–7.91 (m, 1H, 12-H), 7.50–7.37 (m, 6H, 17-H, 22-H, 23-H, 24-H), 7.15–7.03 (m, 2H, 16-H, 18-H), 5.17 (d, J = 4.3 Hz, 2H, 20-H), 3.40–3.33 (m, 2H, 11-H), 3.24 (t, J = 7.6 Hz, 2H, 8-H), 3.21–3.11 (m, 2H, 5-H), 2.70 (dt, J = 11.1,7.0 Hz, 2H, 2-H), 2.07–1.99 (m, 3H, 7-H), 1.61–1.33 (m, 10H, 1-H, 3-H, 4-H, 9-H, 10-H) ppm.

^{13}C NMR (151 MHz, $CDCl_3$, 295 K) δ = 170.2 (C-6), 165.3 (C-13), 156.9 (C 19), 135.7 (C-15), 132.9 (C-17/C-24), 132.5 (C-17/C-24), 129.1 (C-22/C-23), 128.2 (C-22/C-23), 122.1 (C-16/C-18), 112.7 (C-16/C-18), 71.6 (C-20), 48.4 (C-8), 45.3 (C-5), 42.0 (C-2), 39.4 (C-11), 31.0 (C-3/C-4/C-9/C-10), 27.0 (C-3/C-4/C-9/C-10), 26.8 (C-3/C-4/C-9/C-10), 26.5 (C-3/C-4/C-9/C-10), 26.2 (C-3/C-4/C-9/C-10), 25.2 (C-3/C-4/C-9/C-10), 21.6 (C-7) ppm.

HRMS (ESI+) calculated for $[C_{24}H_{33}N_3O_3 + H]^+$ m/z 412.2595, found m/z 412.2601 with a deviation of 1.46 ppm.

Benzyl protected *ortho*-MLL (9)

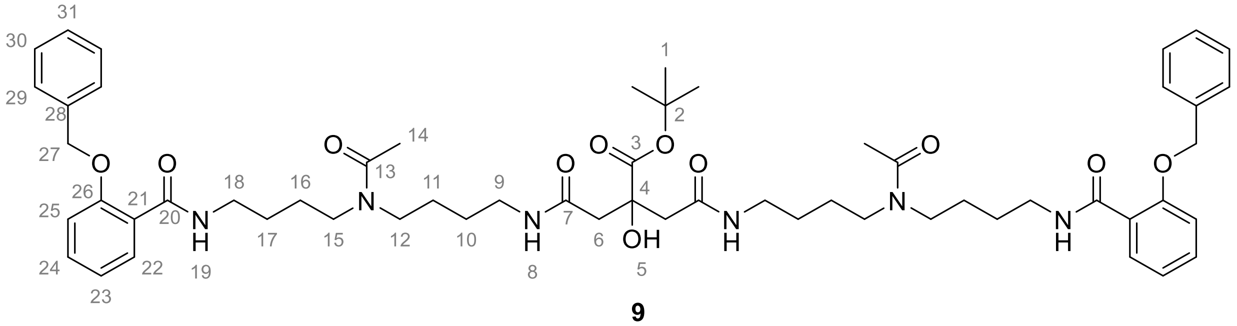

9

The reaction was performed according to a modified literature procedure.[3] In a 25 mL round-bottomed flask, compound **8** (253 mg, 616 µmol, 2.10 equiv.) was dissolved in 3 mL of absolute dichloromethane before adding triethylamine (0.50 mL, 3.62 mmol, 12.3 equiv.) to the solution. Then, compound **4** (130 mg, 293 µmol, 1.00 equiv.) was dissolved in 2 mL of absolute 1,4- dioxane in a 100 mL round-bottomed flask, to which compound **8** was transferred. The remaining 25 mL flask was washed once with 1 mL of absolute dichloromethane and the solution was added to the reaction mixture. After stirring for 15 h under the exclusion of light, LC-MS indicated full conversion of the starting material and the solids were removed by filtration over a cotton plug. After washing with 2 mL of dichloromethane, the volatiles were removed under reduced pressure. The crude yellow oil was dissolved in 15 mL of dichloromethane and washed with 3 x 20 mL of a saturated disodium carbonate. Then, the aqueous layer was extracted with 3 x 20 mL of dichloromethane. The combined organic layers were dried over anhydrous sodium sulfate for 30 min and the volatiles were removed under reduced pressure. The crude product was purified by flash column chromatography (silica; grading from 2 % AM, 98 % DCM to 5 % AM, 95 % DCM to 10 % AM, 90 % DCM) yielding compound **9** (162 mg, 156 µmol, 53 %) as colorless oil.

R_f = 0.4 (UV; 10 % AM, 90 % DCM)

^{1}H NMR (600 MHz, $CDCl_3$, 295 K): δ = 8.23–8.14 (m, 2H, 22-H), 7.95 (d, J = 6.3 Hz, 2H, 19-H), 7.49–7.37 (m, 12H, 24-H, 29-H, 30-H, 31-H), 7.14–7.03 (m, 4H, 23-H, 25-H), 5.17 (d, J = 2.6 Hz, 4H, 27-H), 3.42-3.08 (m, 14H, 9-H 12-H, 15-H, 18-H), 2.75–2.53 (m, 4H,6-H), 2.08–1.96 (m, 6H, 14-H), 1.58–1.33 (m, 18H, 1-H, 10-H, 11-H, 16-H, 17-H) ppm.

^{13}C NMR (151 MHz, $CDCl_3$, 295 K) δ = 172.9 (C-3), 170.3 (C-13), 169.9 (C 7), 165.4 (C-20), 156.9 (C-26), 135.7 (C-28), 133.0 (C-24), 132.4 (C-22), 129.1 (C-30), 128.2 (C-29), 128.1 (C-31), 121.8 (C-23), 121.7 (C-21), 112.7 (C-25), 82.8 (C-2) 74.1 (C-4), 71.5 (C-27),

48.5 (C-9/C-12/C-15), 45.2 (C-9/C-12/C-15), 44.1-43.7 (C-6), 39.3 (C-18), 39.0 (C-9/C-12/C-15), 27.9 (C-1), 26.9 (C-10/C 11/C-16/C-17), 26.7 (C-10/C-11/C-16/C-17), 26.1 (C-10/C-11/C-16/C-17), 25.1 (C-10/C-11/C-16/C-17), 21.6 (C-14) ppm.

HRMS (ESI+) calculated for $[C_{58}H_{78}N_6O_{11} + H]^+$ *m/z* 1035.5801, found *m/z* 1035.5795 with a deviation of −0.579 ppm.

Ortho-MLL

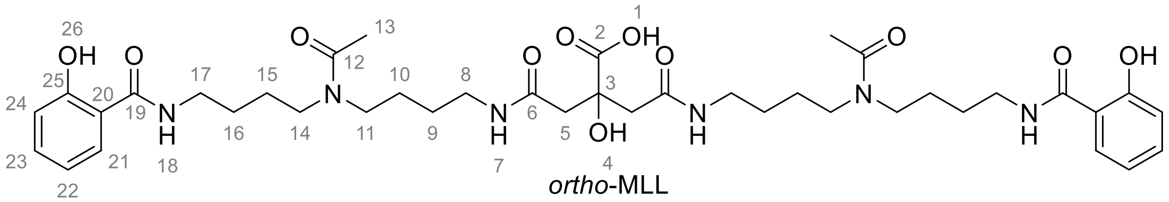

The reaction was performed according to a modified literature procedure.[3] In a 100 mL round-bottomed flask, compound **9** (93.5 mg, 90.3 µmol, 1.00 equiv.) was dissolved in 13 mL of concentrated hydrochloric acid. After stirring for 2.5 h under the exclusion of light, the flask was cooled with an ice bath and 30 mL of saturated sodium bicarbonate solution were added. Then 30 mL of 6 M sodium hydroxide solution was added to reach pH 8. Removing residual inorganic salts from crude *ortho*-MLL was performed using a hydrophilic-lipophilic balance (HLB) column (60 µm, 15 mL/1 g) by *Macherey-Nagel* on a SPE vacuum station. Without running dry, the column was washed with 20 mL of methanol and equilibrated with 20 mL of water, before loading the aqueous crude sample. The loaded column was washed with 35 mL of water and afterwards dried under reduced pressure. Then *ortho*-MLL was eluted with 40 mL of methanol. The two extraction fractions were combined and diluted 1:1 with water. After removing the solvents by lyophilization, a white solid remained as crude product, which was dissolved in 10 mL of methanol and subsequently purified by preparative HPLC (*Dr. Maisch* ReproSil Gold 120 C18 column (250 mM x 10 mm, 5 µm), Eluent A: H_2O + 0.1 % FA, Eluent B: ACN + 0.1 % FA, holding 2 % B for 2 min, gradient to 25 % B in 3 min, to 80 % B in 21 min and to 98 % B in 1 min, flowrate: 13.2 mL/min) yielding ***ortho*-MLL** (33.3 mg, 41.7 µmol, 46 %) as colorless solid.

HPLC: R_t = 16.9 min (254 nm).

^{1}H NMR (600 MHz, D_2O + 0.003 % TMSP-d_4, 295 K): δ = 7.69–7.64 (m, 2H, 21-H), 7.43 (t, J = 7.7 Hz, 2H, 23-H), 7.00–6.94 (m, 4H, 22-H, 24-H), 3.41–3.34 (m, 4H, 17-H), 3.34–3.21 (m, 8H, 11-H, 14-H), 3.09 (q, J = 7.0 Hz, 4H, 8-H), 2.68–2.55 (m, 4H, 5-H), 2.06 (t, J = 14.5 Hz, 6H, 13-H), 1.66–1.33 (m, 16H, 9-H, 10-H, 15-H, 16-H) ppm.

^{13}C NMR (151 MHz, D_2O + 0.003 % TMSP-d_4, 295 K) δ = 181.7 (C-2), 176.4 (C-12), 174.9 (C-6), 172.6 (C-19), 160.2 (C-25) 137.0 (C-23), 131.2 (C-21), 123.0 (C-22/C-24), 120.1 (C-22/C-24), 77.8 (C-1), 51.7 (C-11/C-14/C-17), 48.6 (C-11/C-14/C-17), 47.2 (C-5), 41.9 (C-

11/C-14/C-17), 41.7 (C-8), 28.7 (C-7/C-8/C-13/C-14), 28.5 (C-7/C-8/C-13/C-14), 28.1 (C-7/C-8/C-13/C-14), 27.2 (C-9/C-10/C-15/C-16), 23.4 (C-13) ppm.

HRMS (ESI+) calculated for $C_{40}H_{59}N_6O_{11}$ $[M+H]^+$ *m/z* 799.4236, found *m/z* 799.4237 with a deviation of 0.125 ppm.

^{1}H NMR of compound **2** in $CDCl_3$ (600 MHz)

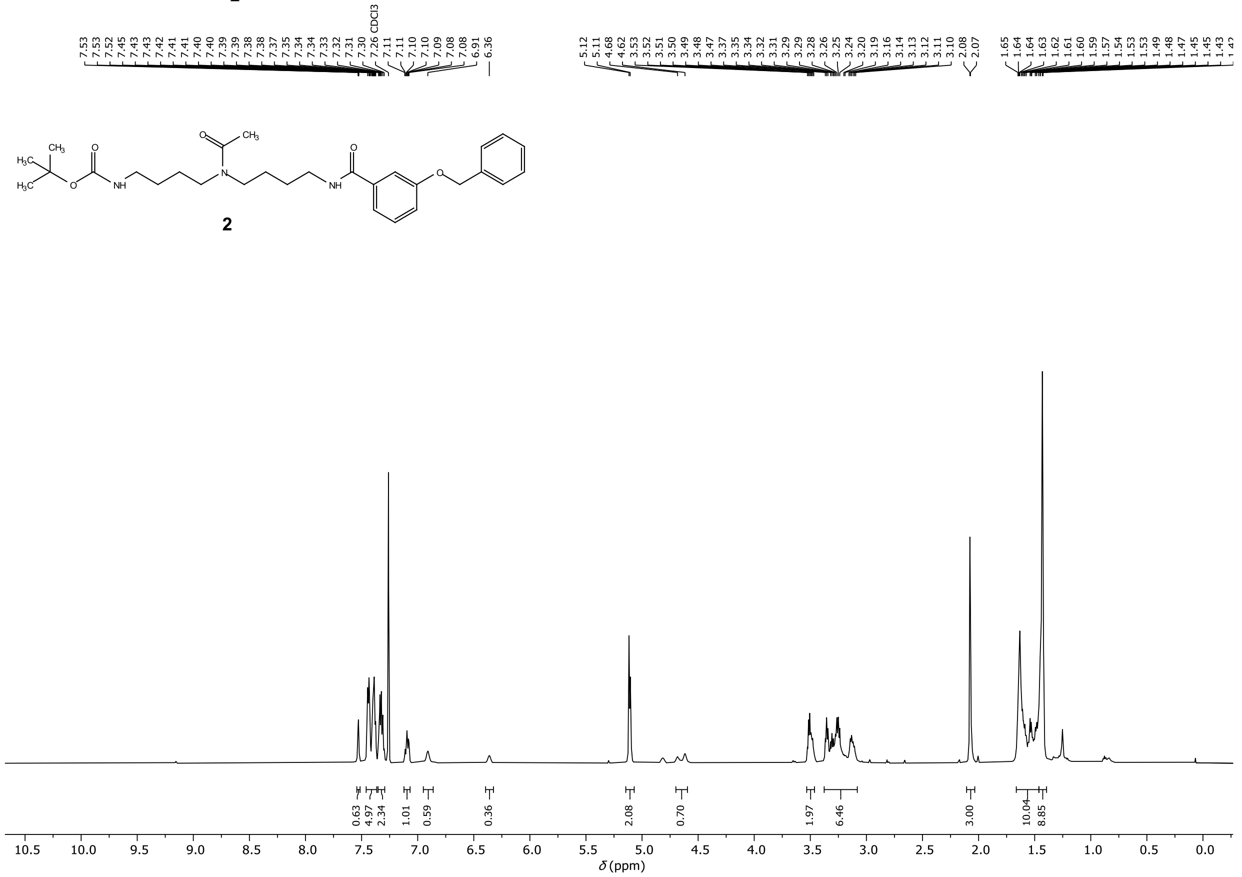

^{13}C NMR of compound **2** in $CDCl_3$ (151 MHz)

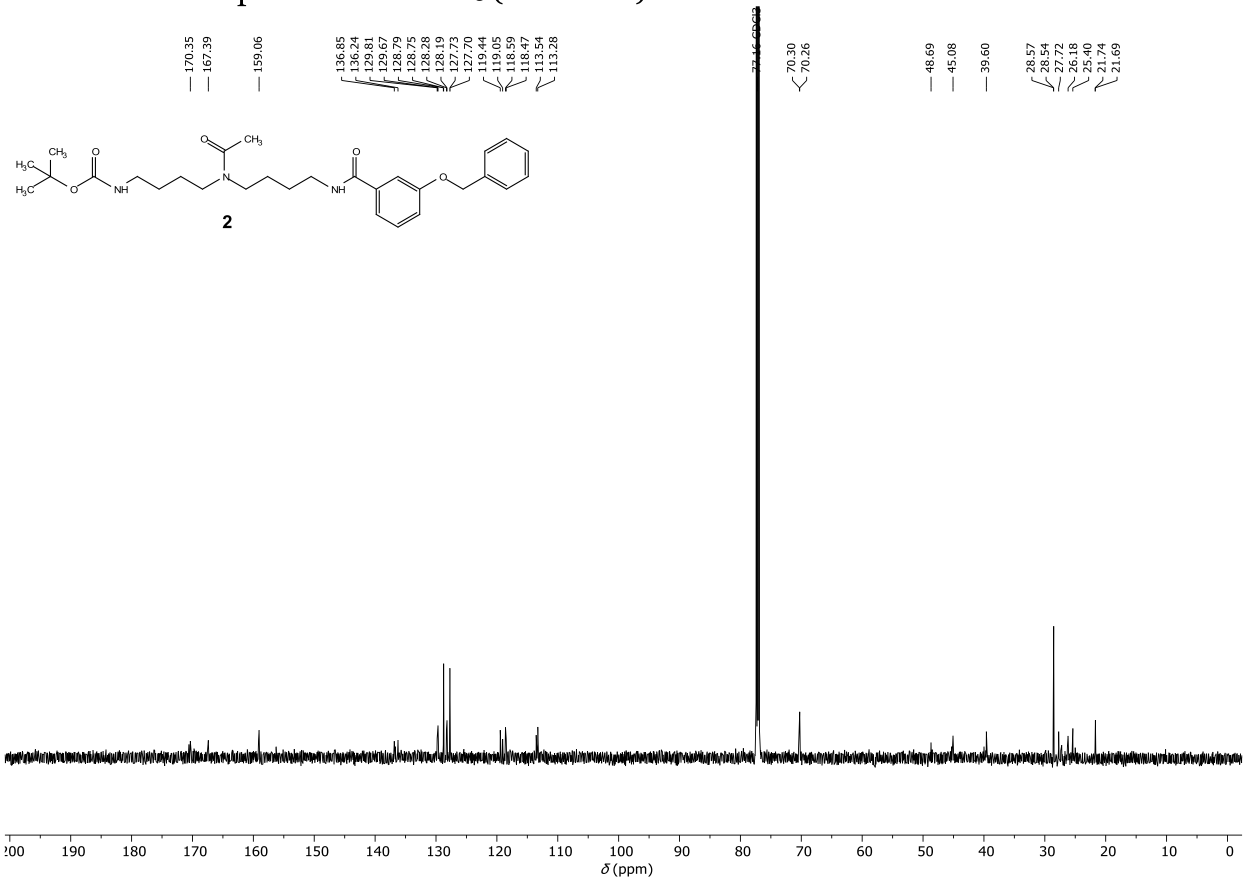

^{1}H NMR of compound **3** in $CDCl_3$ (600 MHz)

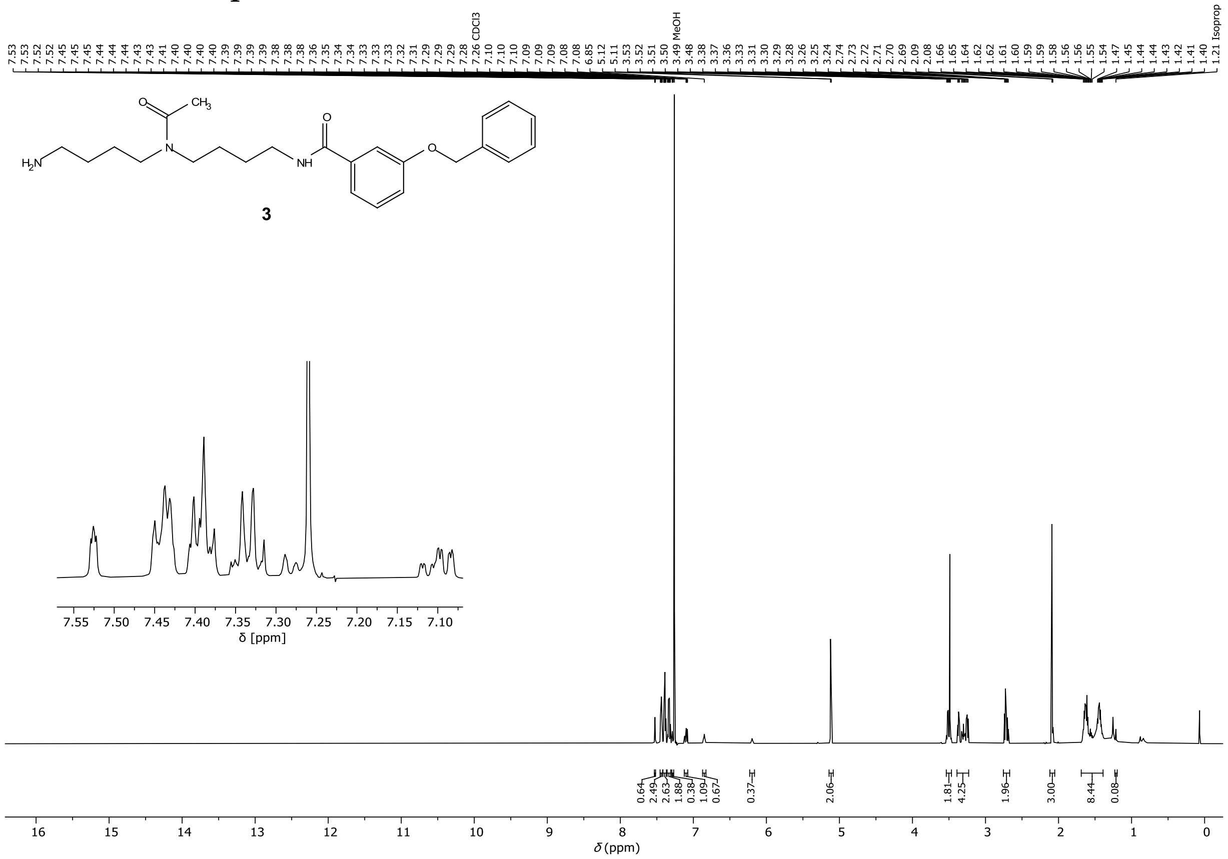

^{13}C NMR of compound **3** in $CDCl_3$ (151 MHz)

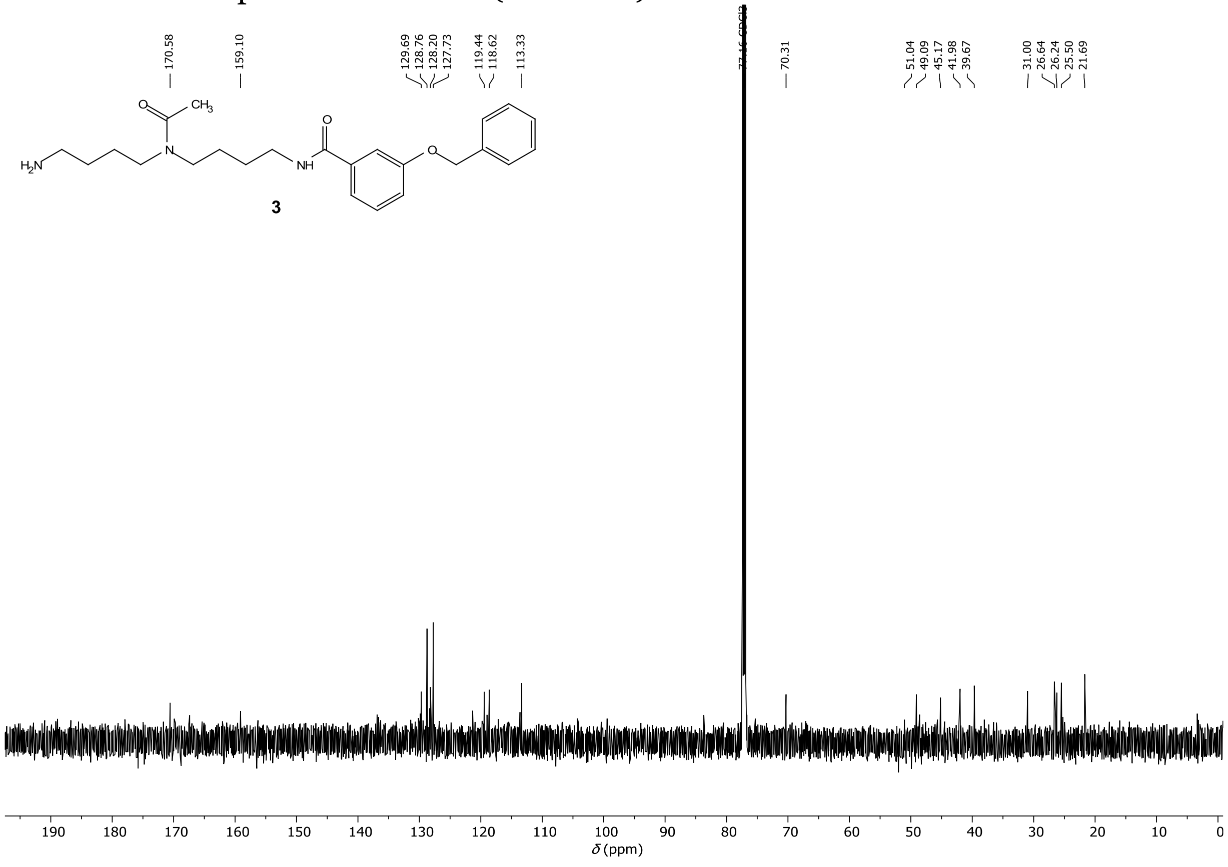

^{1}H NMR of compound **5** in $CDCl_3$ (600 MHz)

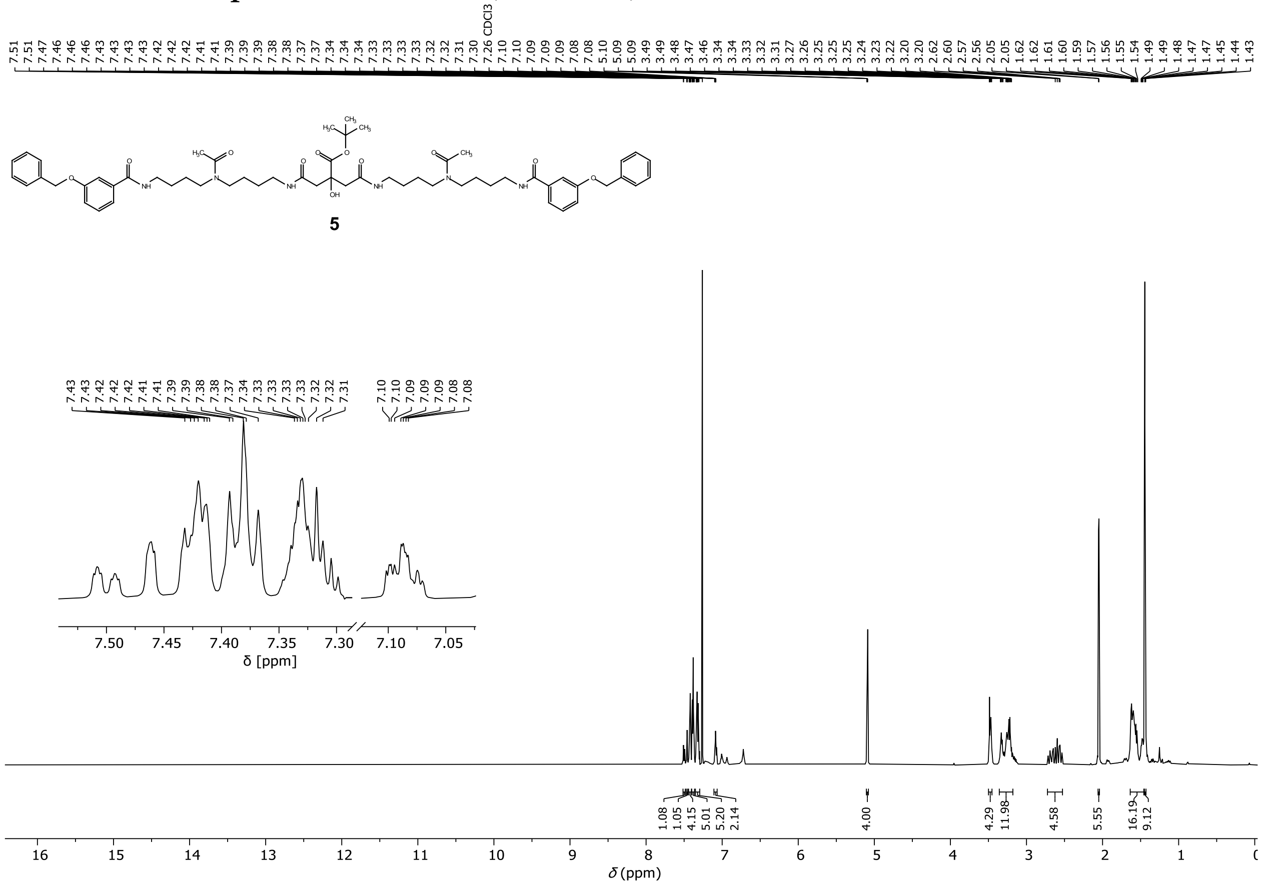

^{13}C NMR of compound **5** in $CDCl_3$ (151 MHz)

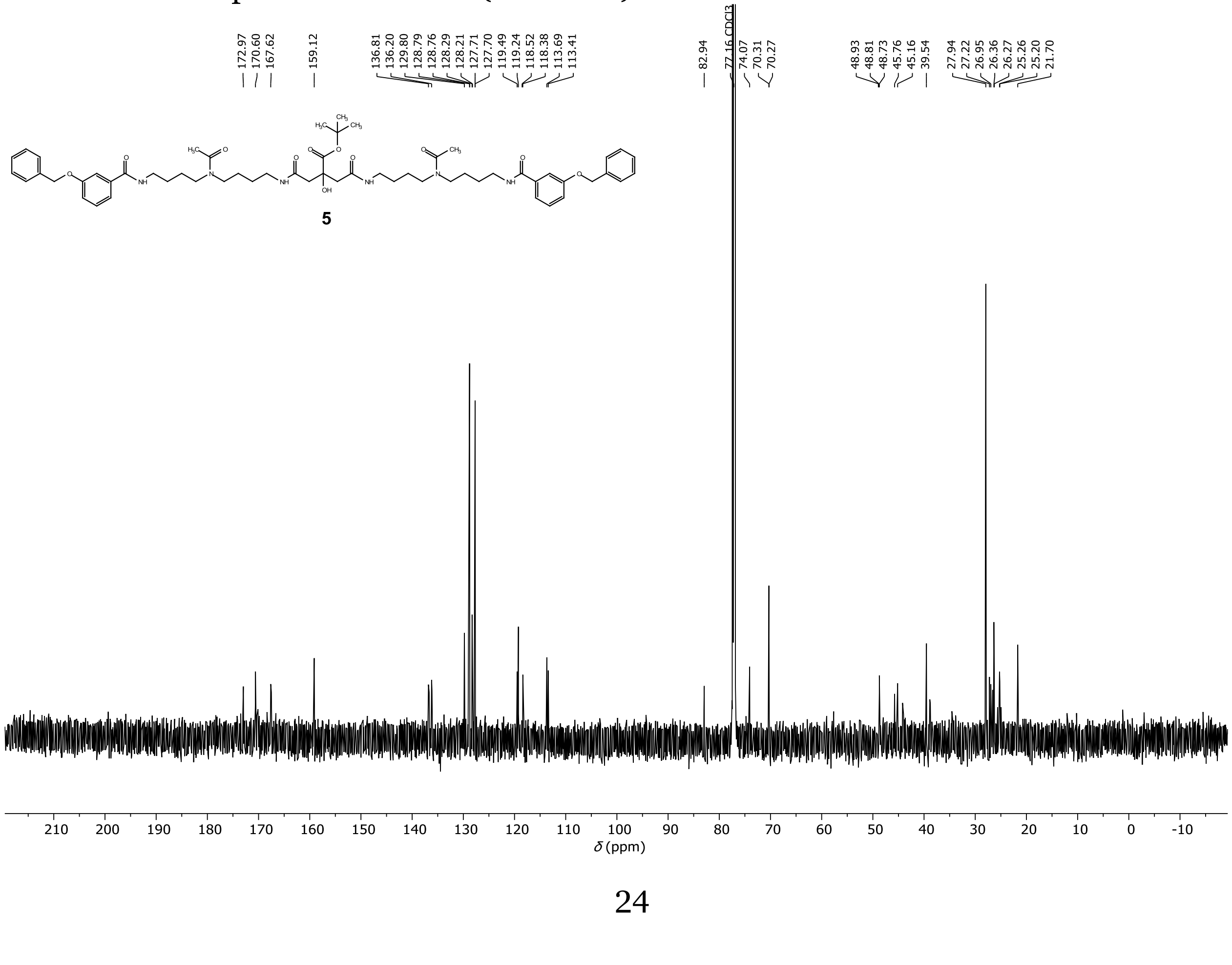

^{1}H NMR of *meta*-MLL in H_2O/D_2O + 0.003% TMSP-*d*$_4$ (9:1, 600 MHz)

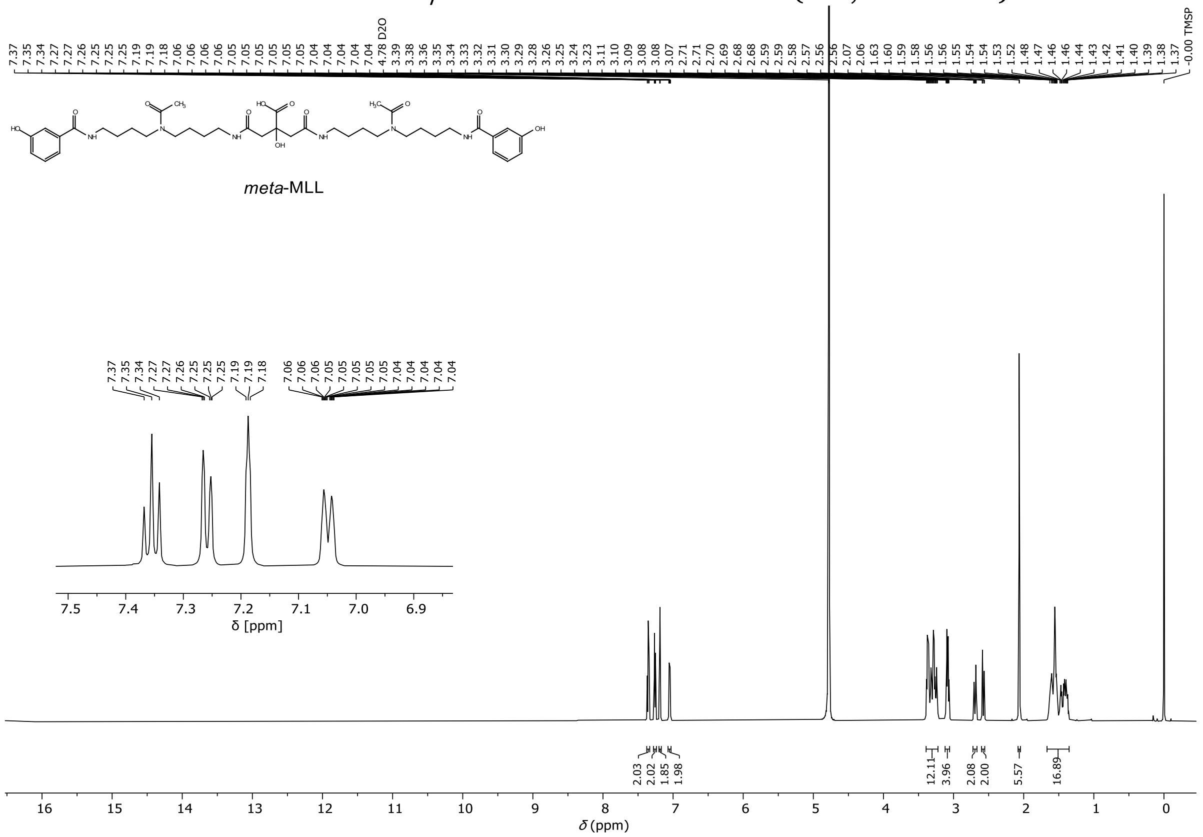

^{13}C NMR of *meta*-MLL in H_2O/D_2O + 0.003% TMSP-*d*$_4$ (9:1, 151 MHz)

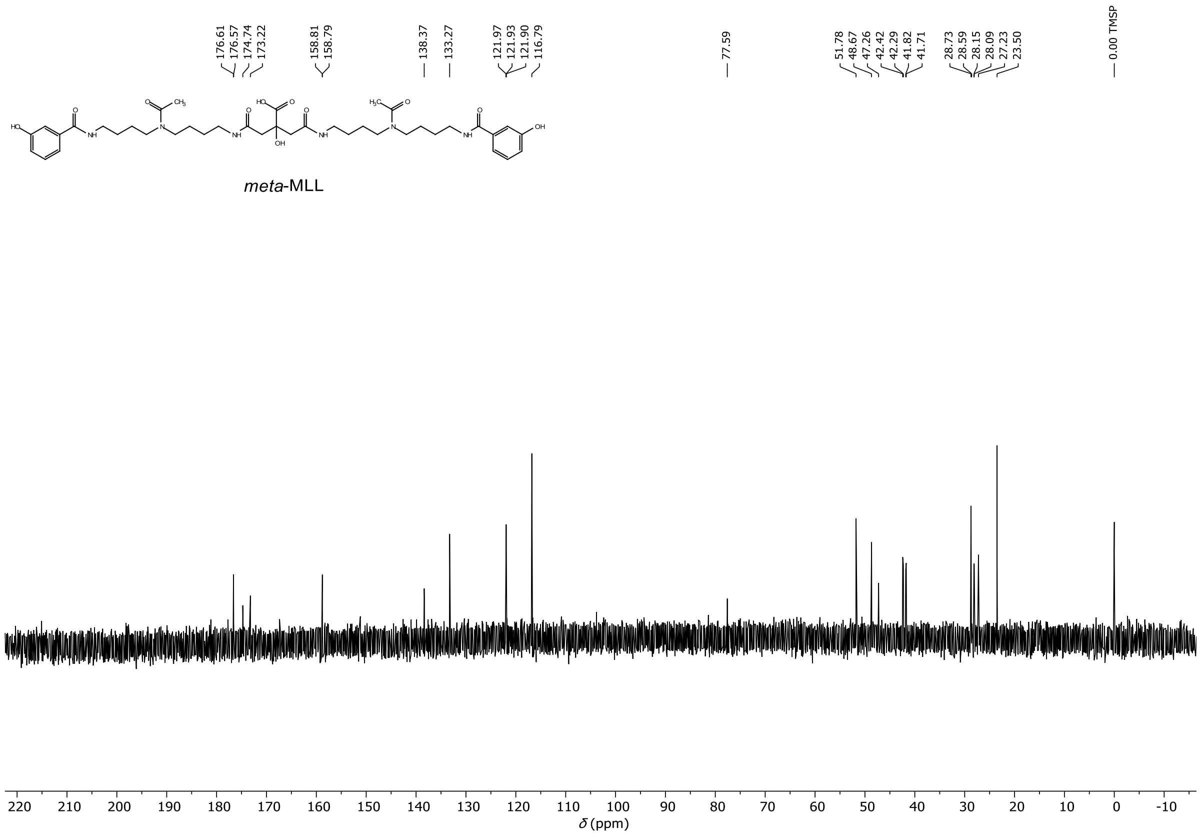

^{1}H NMR spectrum of compound **7** in $CDCl_3$ (600 MHz)

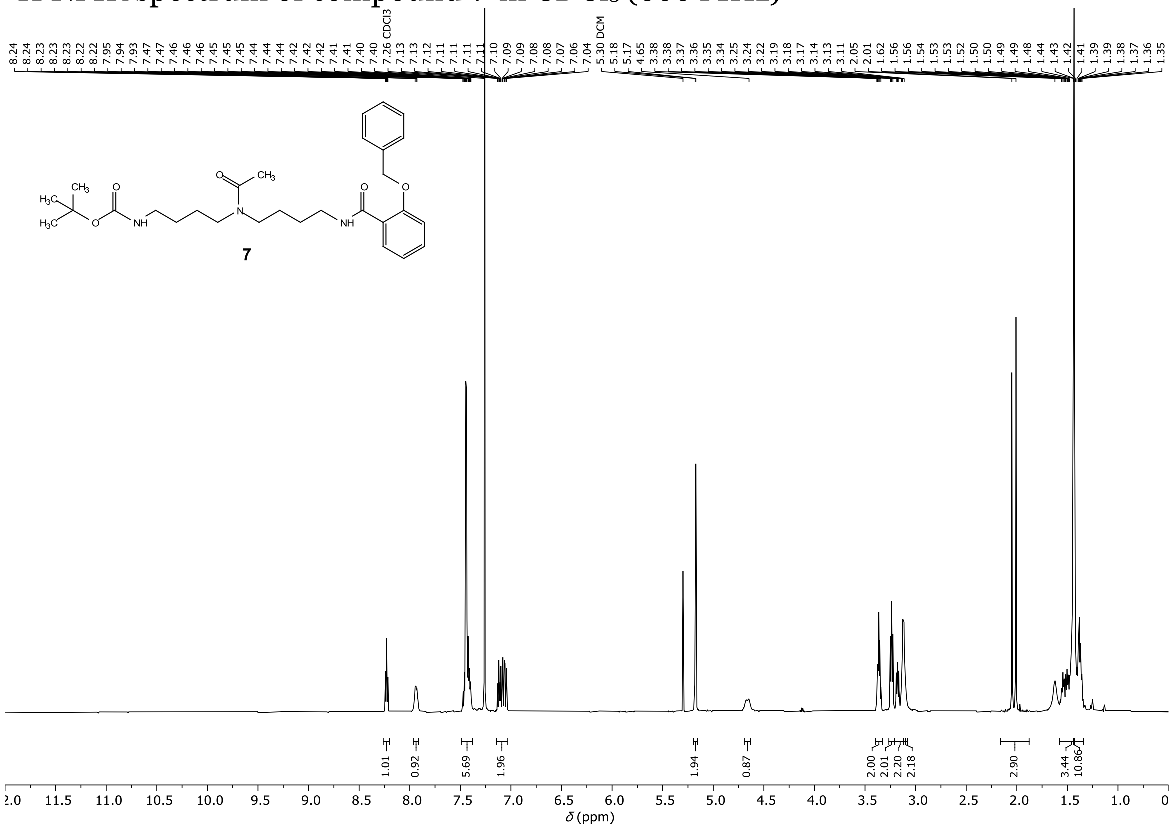

^{13}C NMR spectrum of compound **7** in $CDCl_3$ (151 MHz)

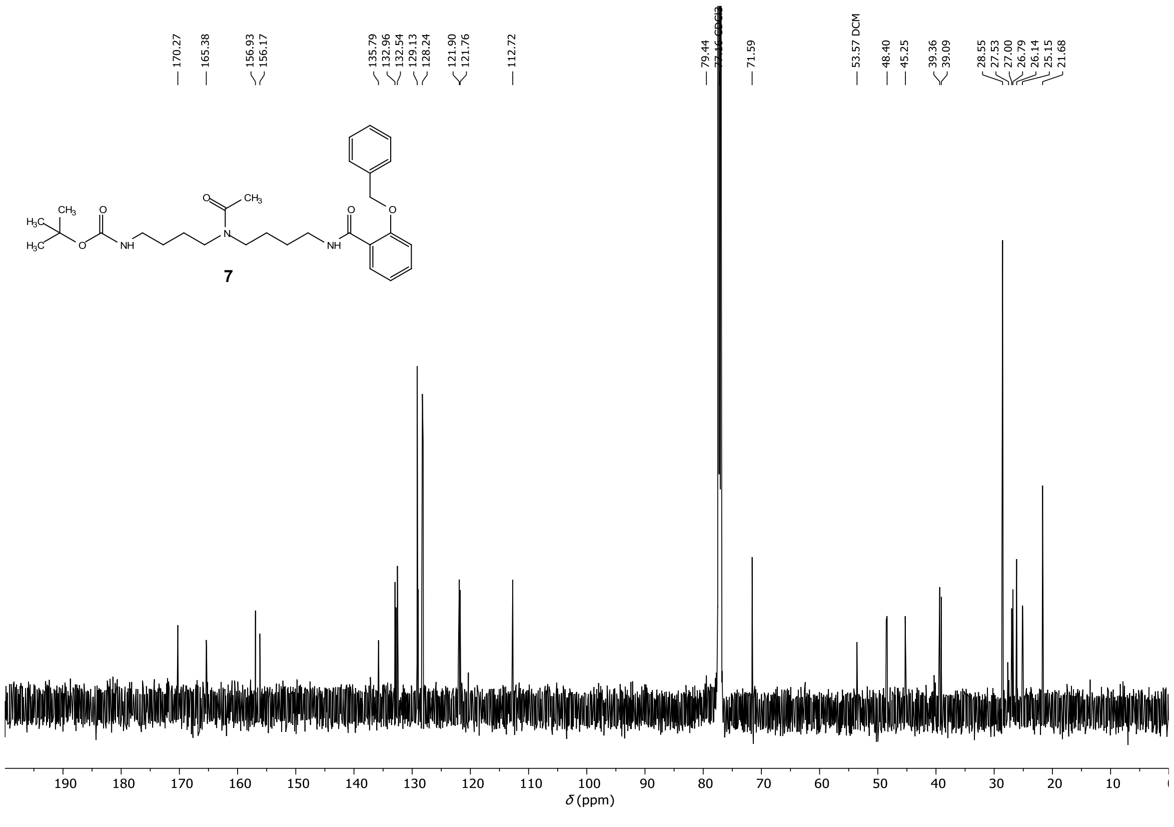

^{1}H NMR spectrum of compound **8** in $CDCl_3$ (600 MHz)

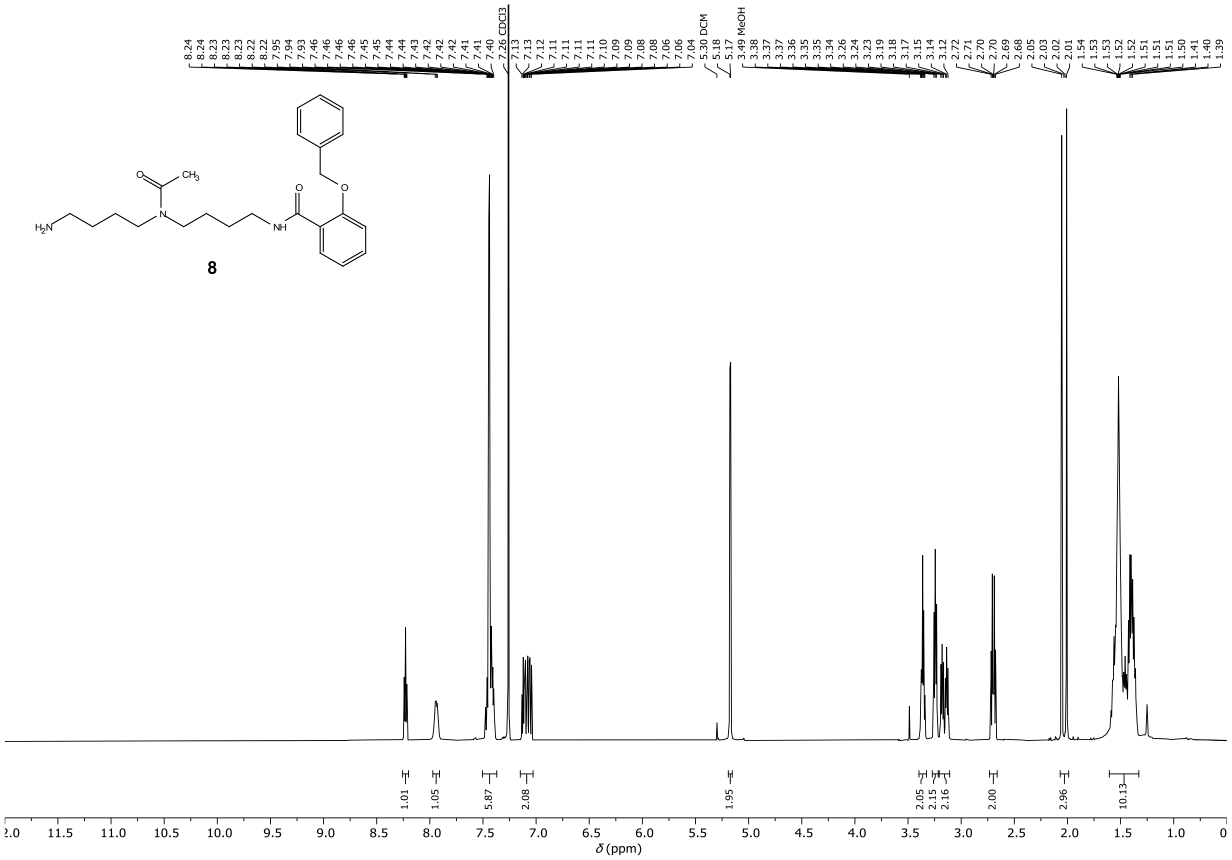

^{13}C NMR spectrum of compound **8** in $CDCl_3$ (151 MHz)

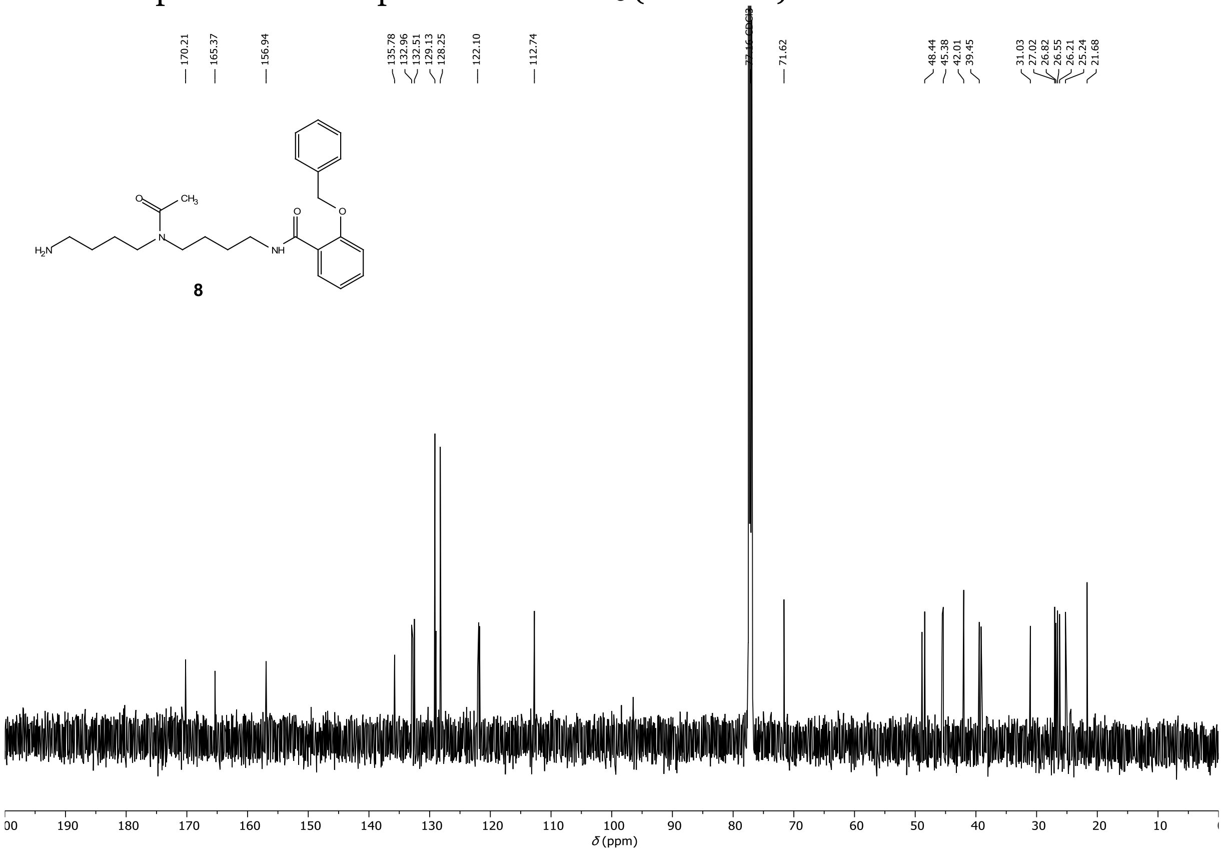

^{1}H NMR spectrum of compound **9** in $CDCl_3$ (600 MHz)

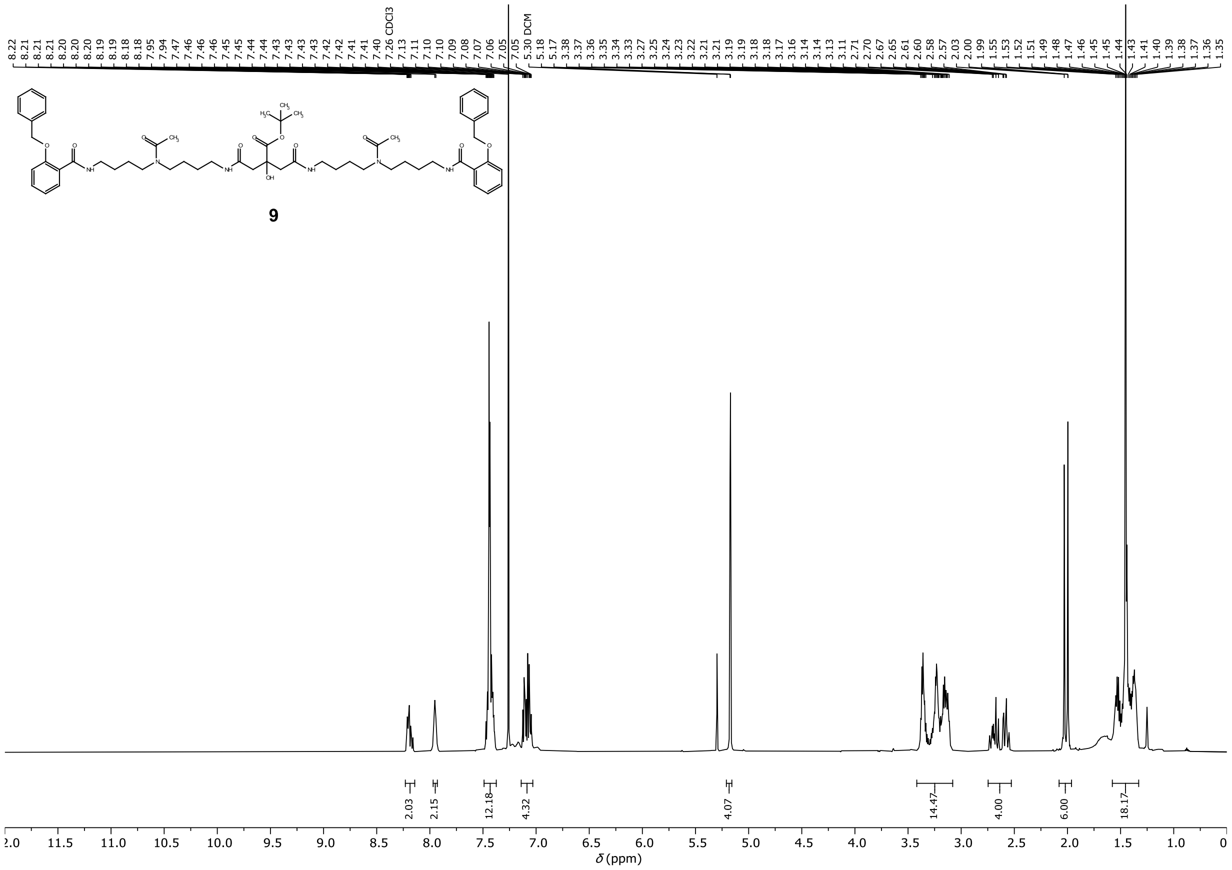

^{13}C NMR spectrum of compound **9** in $CDCl_3$ (151 MHz)

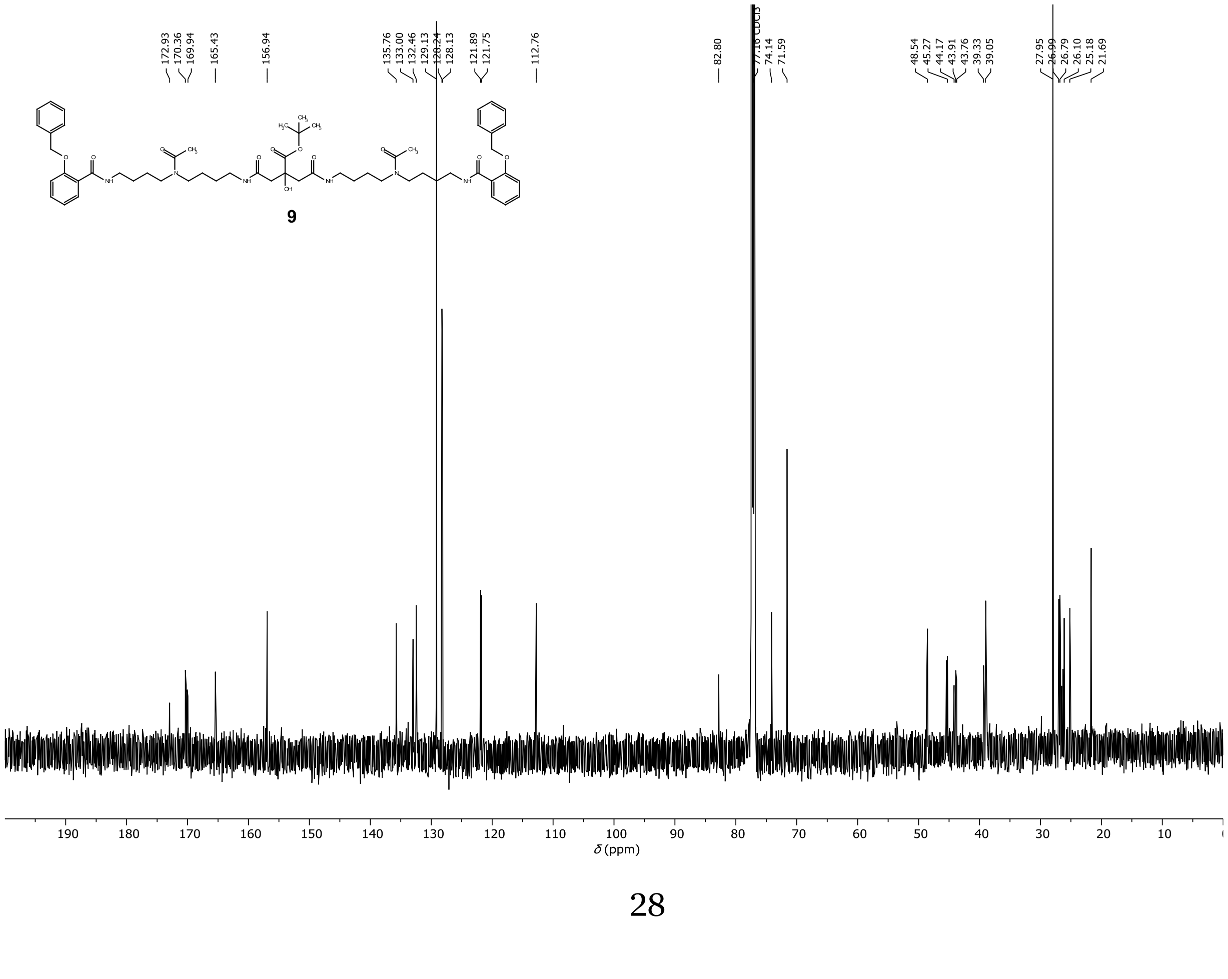

^{1}H NMR of *ortho*-Methylolanthanin in D_2O + 0.003 % TMSP-d_4 (600 MHz)

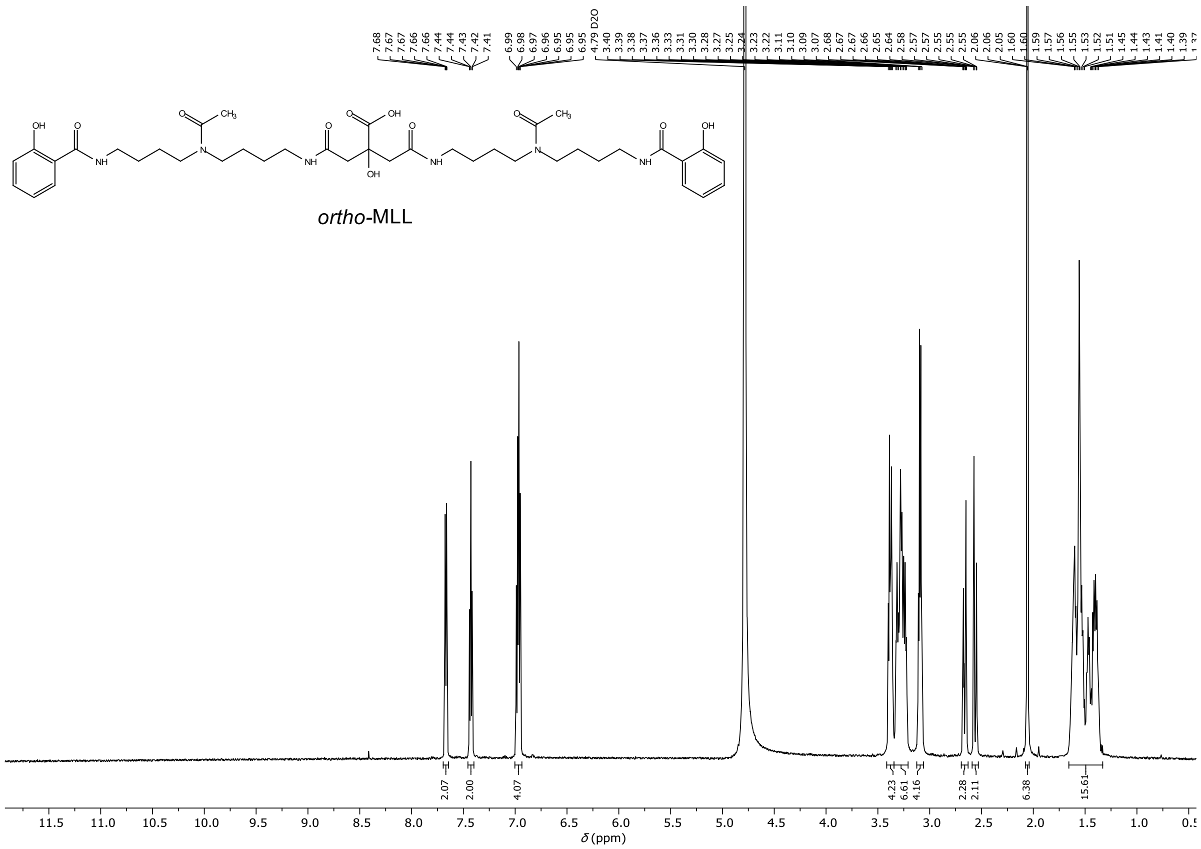

^{13}C NMR spectrum of *ortho*-Methylolanthanin D_2O + 0.003 % TMSP- d_4 (151 MHz)

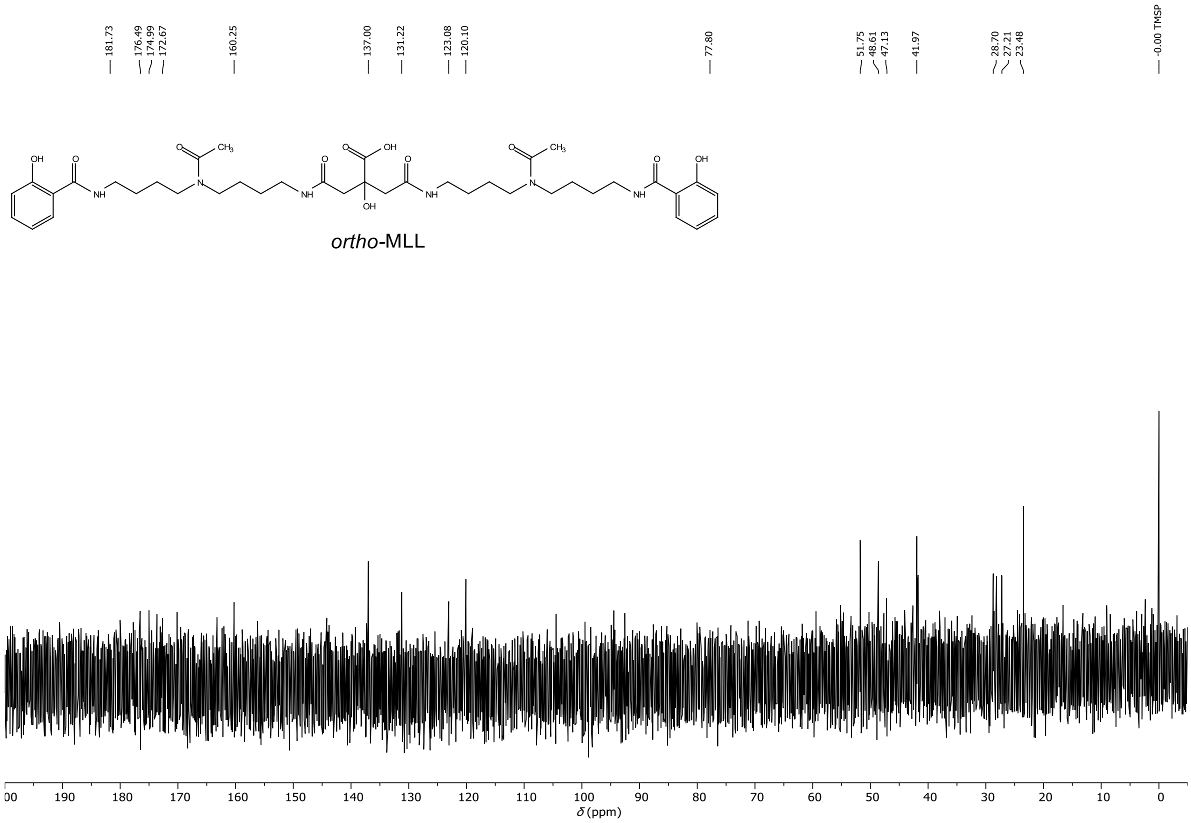

3. Binding Studies

Lanthanide to Ligand Titration Experiments

The titration experiments were conducted according to a literature procedure.[3]

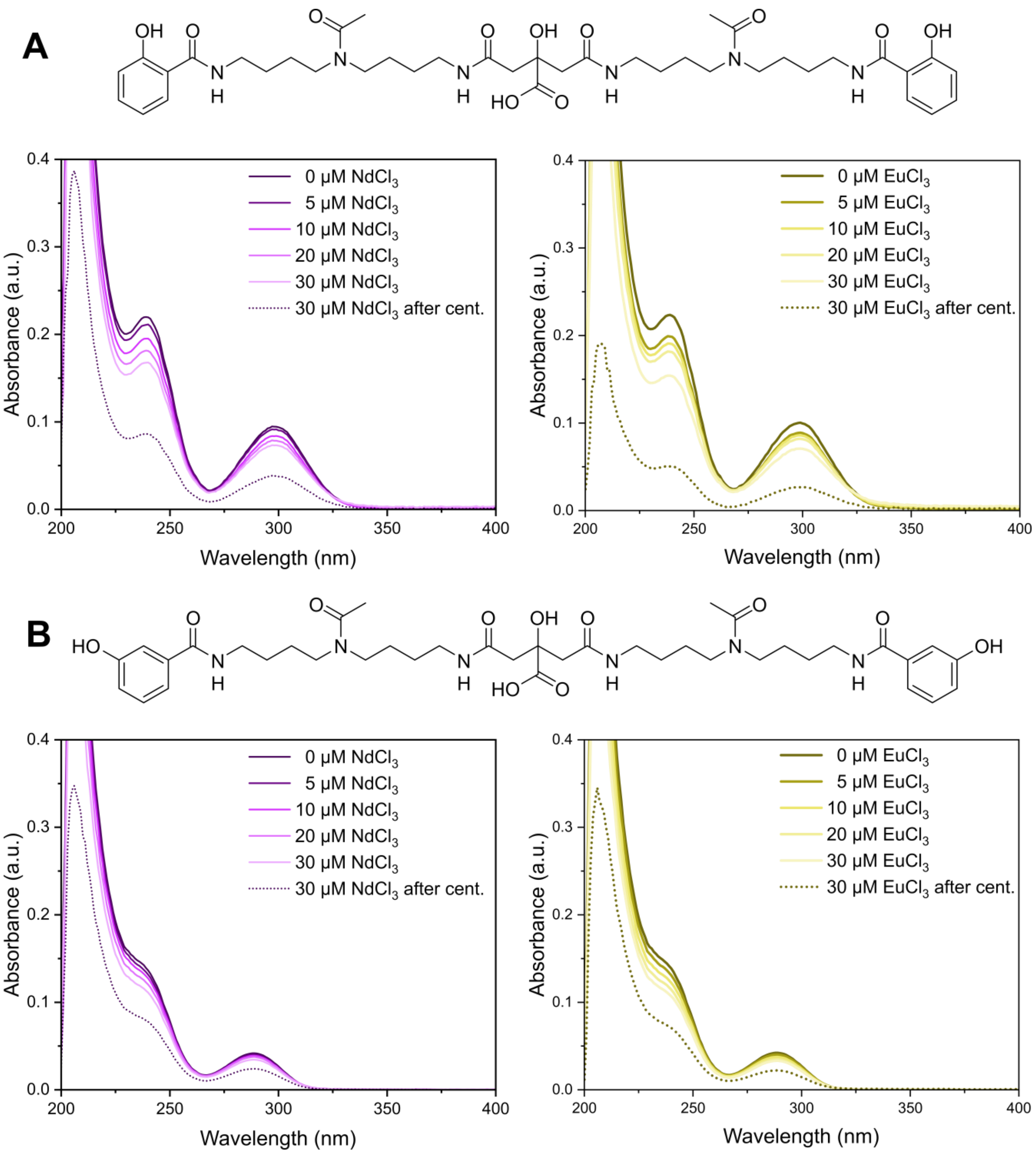

Figure S3: UV-vis spectra of $LnCl_3$ (Ln = Nd, Eu) to x-MLL (10 µM) titrations in buffer (10 mM MOPSO, 100 mM KCl, pH 6.0). Titrations were performed over four steps with final Ln^{3+} concentrations ranging from 0 µM to 30 µM. The 30 µM samples were remeasured after centrifugation (21,000 × g, 15 minutes, 25 °C). ***A*** *represents the data for* ortho-*MLL and* ***B*** *for* meta-*MLL.*

Chrome Azurol S (CAS) Fe^{3+} Competition Experiment

The CAS competition experiments were conducted according to a previously described procedure.[3]

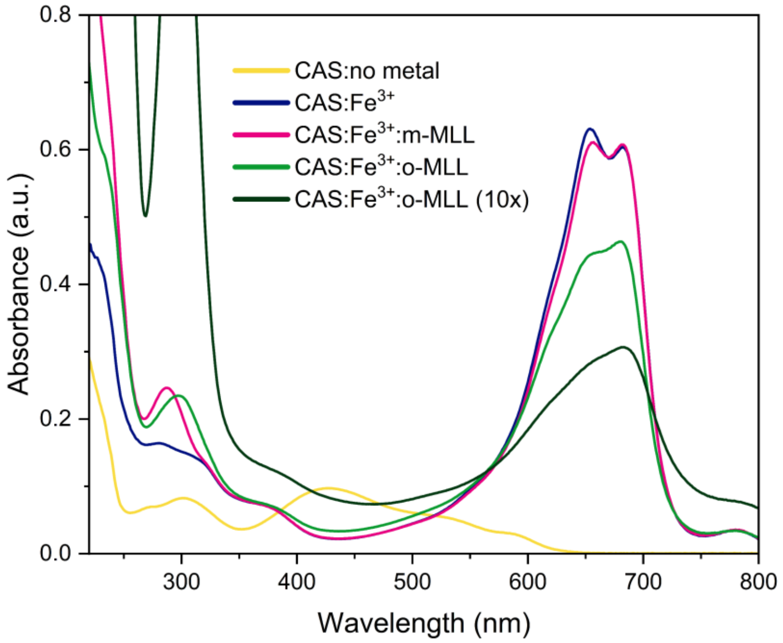

Figure S4: CAS competition experiment of ortho-/meta-*MLL in MOPSO buffer (10 mM, 100 mM KCl, pH 6.0) with $FeCl_3$ (final concentration: CAS/ HDTMA (18.75 µM/ 200 µM), $FeCl_3$ (18.75 µM), ligand (18.75 µM) or excess ligand (187.50 mM).*

Nitrilotriacetic acid (NTA) Eu^{3+} Competition Experiment

The TRLFS experiments were conducted and analysed according to a modified literature procedure.[3] All conditions were kept constant with the exception of the respective nitrilotriacetic acid (NTA) concentrations, which were changed to 0.0, 1.0, 1.5 or 2.0 µM, respectively. In total this experiment involved 62 samples, due to a common control series without MLL, which are represented in Table S1.

Table S1: Sample overview of the TRLFS competition experiments.

Sample	Series #	Subset	Description
1-8	1	1-4<br>5-8	Series 1 centrifuged + acidified<br>Series 1 centrifuged
9-16	2	9-12<br>13-16	Series 2 centrifuged + acidified<br>Series 2 centrifuged
17-24	3	17-20<br>21-24	Series 3 centrifuged + acidified<br>Series 3 centrifuged
25-27	1-3	0 µM NTA samples	Samples without centrifugation
28-35	4 (Control)	28-31<br>32-35	Control Series without centrifugation<br>Control Series acidified

PARAFAC analysis with integrated speciation has proven to be a robust approach for interpretation of TRLFS data, enabling the assignment of spectroscopic components to distinct chemical species.[5,6] The PARAFAC results for the aforementioned sample sets are presented in Figures S5 and S6.

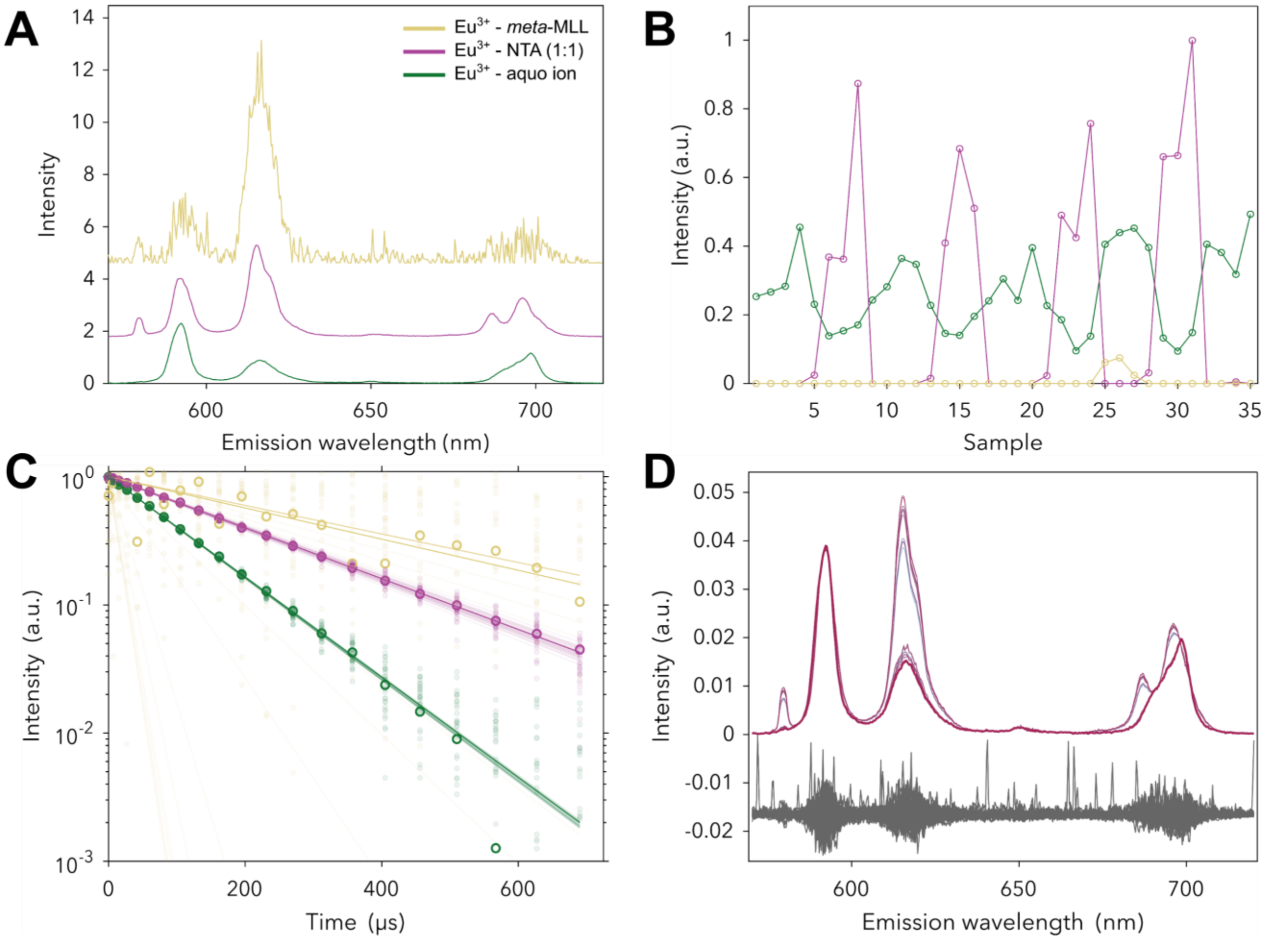

Figure S5: PARAFAC results of the sample set listed in Table S1 from meta-*MLL. (**A**) Stacked emission spectra of the observed species within the sample set and (**B**) sample distribution corresponding to those listed in Table S1. Due to precipitation, only in the samples without centrifugation (25-27) the Eu^{3+}-MLL complex can be detected. (**C**) Luminescence lifetimes of the respective species and (**D**) data versus noise.*

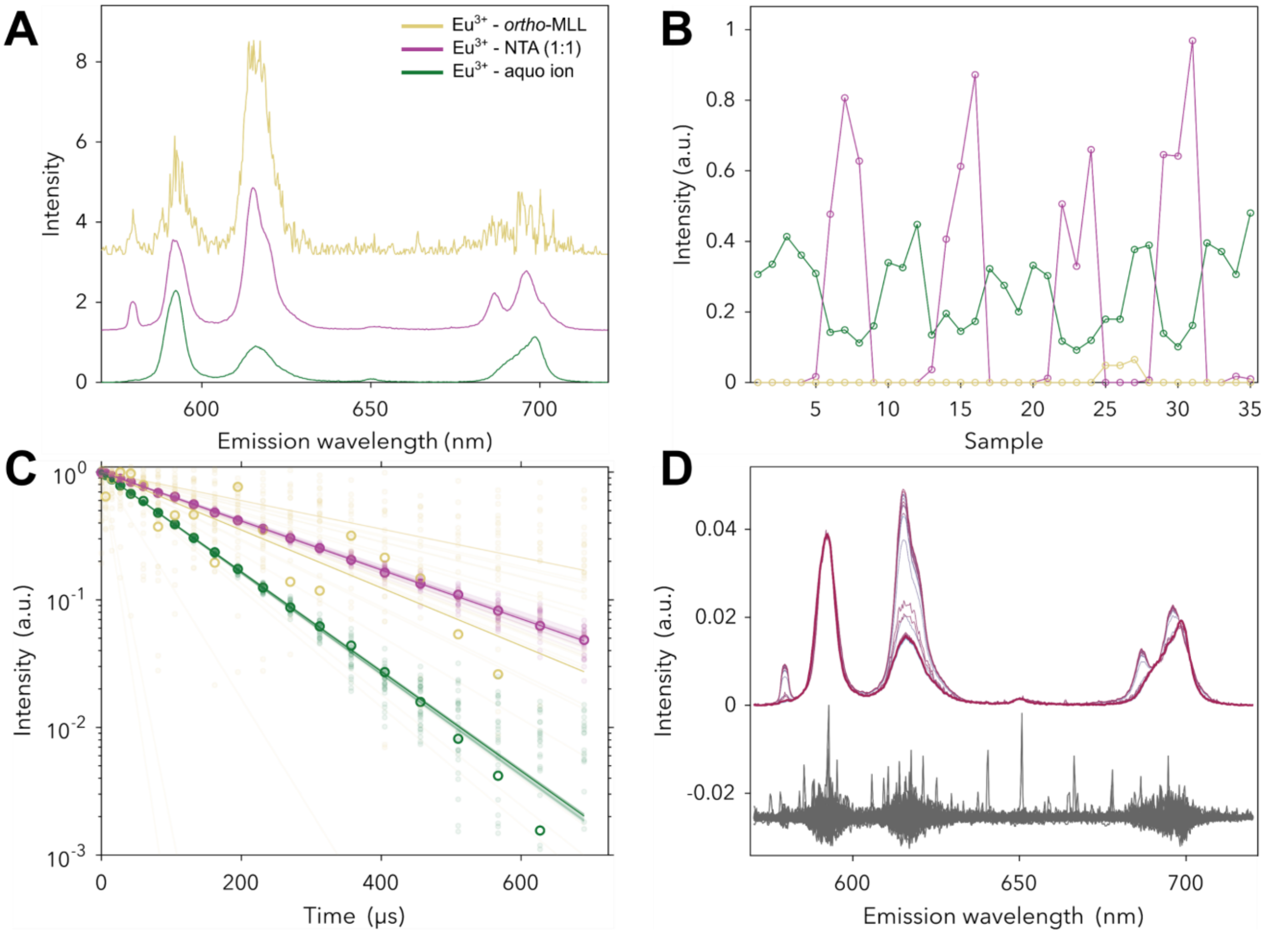

Figure S6: PARAFAC results of the sample set listed in Table S1 from ortho-*MLL. (**A**) Stacked emission spectra of the observed species within the sample set and (**B**) sample distribution corresponding to those listed in Table S1. Due to precipitation, only in the samples without centrifugation (25-27) the Eu^{3+}-MLL complex can be detected. (**C**) Luminescence lifetimes of the respective species and (**D**) data versus noise.*

4. Quantum Chemical Calculations

For the quantum chemical calculations we used the same methods as outlined in our previous publication.[3] In brief, candidate structures of the respective ions (dications, either doubly protonated or one proton replaced by La^{3+} or Lu^{3+}, respectively) were obtained in two steps: First, the conformational space was explored with the CREST package,[7] based on the semiempirical XTB program.[8] Then, reasonable structures were optimised with the DFT method as implemented in the TURBOMOLE package.[9,10] Based on our experiences with *para*-MLL,[3] all geometry optimizations were performed with the def-SV(P) basis set[11] and the BP-86 functional.[12-14] In the case of Fe^{3+} we investigated singlet, triplet and quintet multiplicities, where in all cases the high-spin-state is energetically favoured. With the DFT-optimised structures we performed trajectory method (TM) calculations as implemented in the IMoS package.[15,16] In these calculations a large number of collisions between nitrogen molecules (the buffer gas in the drift cell) and the drifting ion are simulated. By integration of the scattering angles the theoretical CCS is obtained that can be compared with the experimental value in order to confirm or rule out a candidate structure. The simulations were based on Lennard-Jones type and ion-induced dipole interactions between the ion and N_2. The partial atomic charges (for the ion-induced dipole interactions) were obtained with a Mulliken population analysis. Deviations between experiment and theory of 2 – 7 % are common.[17] The relative energies and calculated CCS of the geometry optimized structures are summarized in Table S2 (*meta*-MLL) and S3 (*ortho*-MLL) (up to 1 eV above the lowest energy structure). The lowest energy structures are depicted in Figure S7 and S8.

*Table S2: Relative energies and calculated CCS of geometry optimized candidate structures of [*meta-*MLL–H+M]$^{2+}$, M = 3H^+, La^{3+}, Lu^{3+}, Fe^{3+}.*

[*meta*-MLL+2H]$^{2+}$			[*meta*-MLL−H+La]$^{2+}$			[*meta*-MLL−H+Lu]$^{2+}$			[*meta*-MLL−H+Fe]$^{2+}$		
#	relative energy (eV)	CCS (Å^2)	#	relative energy (eV)	CCS (Å^2)	#	relative energy (eV)	CCS (Å^2)	#	relative energy (eV)	CCS (Å^2)
1	0.00	357.7	1	0.00	323.7	1	0.00	317.1	1	0.00	314.8
2	0.08	365.0	2	0.01	319.7	2	0.13	316.6	2	0.25	317.4
3	0.49	358.4	3	0.19	317.6	3	0.16	310.0	3	0.33	310.9
4	0.93	316.8	4	0.21	315.0	4	0.63	348.4	4	0.38	307.8
			5	0.81	321.8	5	0.80	317.0	5	0.67	341.8

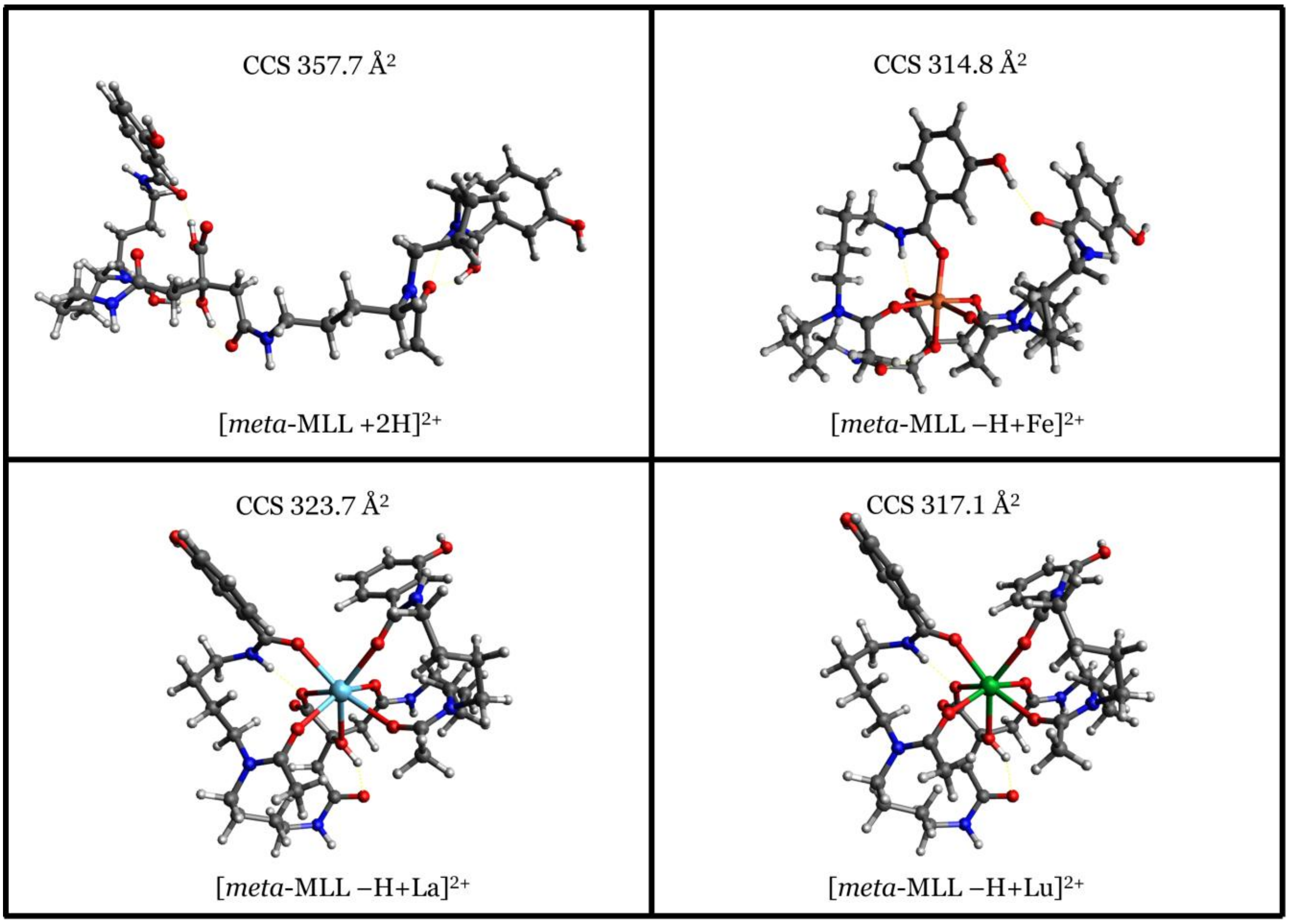

*Figure S7: DFT calculations of [*meta*-MLL−H+M]$^{2+}$ (M = $3H^+$, La^{3+}, Lu^{3+}, Fe^{3+}) complexes. Lowest energy geometries with their corresponding CCS values (see Table S2).*

*Table S3: Relative energies and calculated CCS of geometry optimized candidate structures of [*ortho*-MLL−H+M]$^{2+}$, M = $3H^+$, La^{3+}, Lu^{3+}, Fe^{3+}.*

[*ortho*-MLL+2H]$^{2+}$			[*ortho*-MLL−H+La]$^{2+}$			[*ortho*-MLL−H+Lu]$^{2+}$			[*ortho*-MLL−H+Fe]$^{2+}$		
#	relative energy (eV)	CCS (Å^2)	#	relative energy (eV)	CCS (Å^2)	#	relative energy (eV)	CCS (Å^2)	#	relative energy (eV)	CCS (Å^2)
1	0.00	357.6	1	0.00	318.3	1	0.00	314.6	1	0.00	321.7
2	0.12	361.6	2	0.14	317.7	2	0.23	307.4	2	0.53	336.5
3	0.49	356.7	3	0.45	312.3	3	0.33	305.1	3	0.71	322.3
4	0.70	311.6	4	0.50	318.4	4	0.44	312.1	4	0.76	303.9
			5	0.93	313.2	5	0.55	308.9	5	0.88	316.9
						6	0.74	343.2	6	0.99	338.1

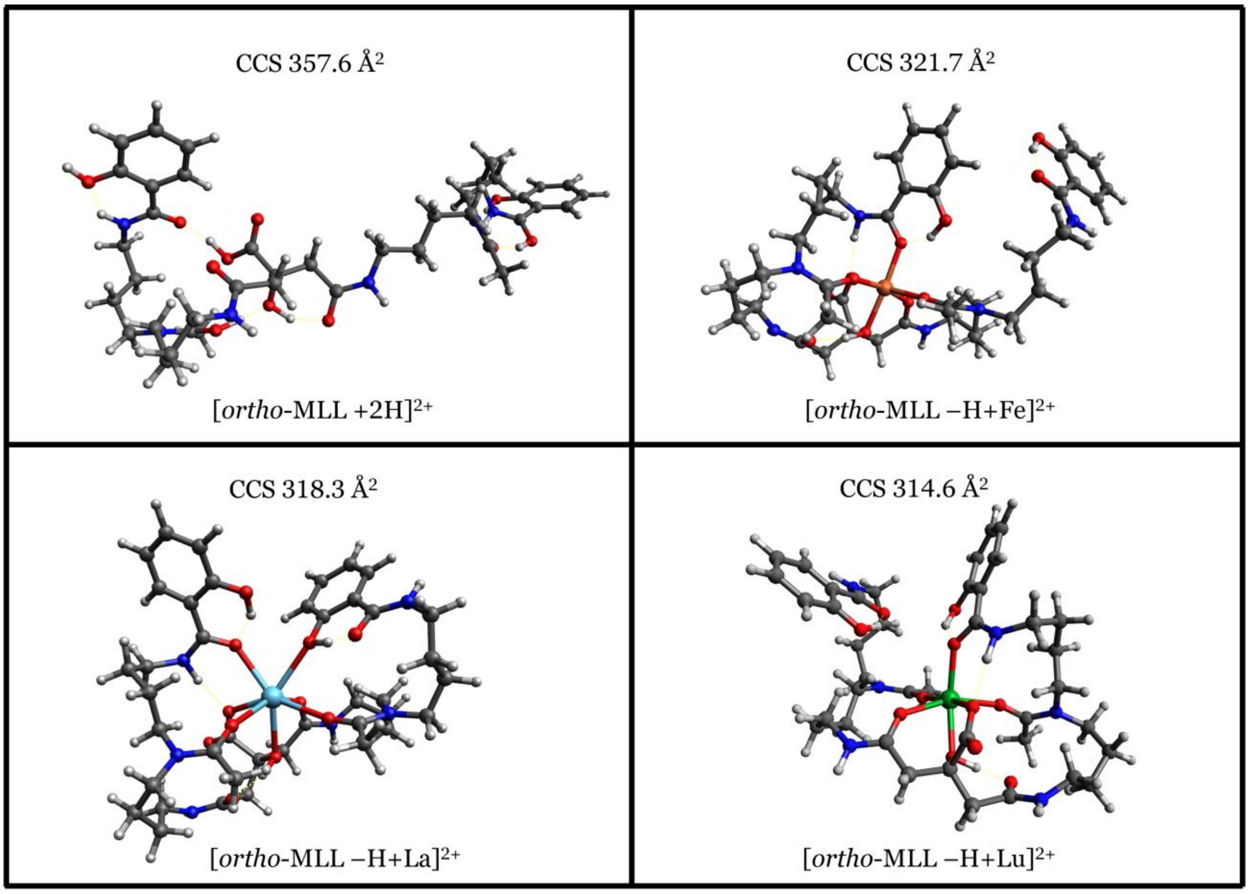

*Figure S8: DFT calculations of [*ortho*-MLL–H+M]$^{2+}$ (M = $3H^+$, La^{3+}, Lu^{3+}, Fe^{3+}) complexes. Lowest energy geometries with their corresponding CCS values (see Table S3).*

5. Data Availability Statement

Primary data concerning UV-vis (.csv), TRLFS (.sif), NMR (Bruker raw data) and quantum chemical calculations (.xyz) shown in this Supporting Information are available at the RODARE repository: https://doi.org/10.14278/rodare.4748.

6. References

1 Zytnick, A. M. *et al.* Identification and characterization of a small-molecule metallophore involved in lanthanide metabolism. *Proc. Natl. Acad. Sci. U. S. A.* **121**, e2322096121 (2024). https://doi.org/doi:10.1073/pnas.2322096121

2 Hennrich, F. *et al.* Cyclic ion mobility of doped [MAu24L18]2– superatoms and their fragments (M = Ni, Pd and Pt; L = alkynyl). *Phys. Chem. Chem. Phys.* **26**, 8408-8418 (2024). https://doi.org/10.1039/D3CP06192B

3 Gutenthaler-Tietze, S. M. *et al.* Comparative Binding Studies of the Chelators Methylolanthanin and Rhodopetrobactin B to Lanthanides and Ferric Iron. *ChemBioChem* **27**, e202500312 (2025). https://doi.org/10.1002/cbic.202500312

4 Stow, S. M. *et al.* An Interlaboratory Evaluation of Drift Tube Ion Mobility–Mass Spectrometry Collision Cross Section Measurements. *Anal. Chem.* **89**, 9048-9055 (2017). https://doi.org/10.1021/acs.analchem.7b01729

5 Drobot, B. *et al.* Speciation Studies of Metals in Trace Concentrations: The Mononuclear Uranyl(VI) Hydroxo Complexes. *Anal. Chem.* **88**, 3548-3555 (2016). https://doi.org/10.1021/acs.analchem.5b03958

6 Lopez-Odriozola, L., Shaw, S., Abrahamsen-Mills, L., Waters, C. & Natrajan, L. S. Identification and Quantification of Multiphase U(VI) Speciation on Gibbsite with pH Using TRLFS and PARAFAC of Excitation Emission Matrices. *Environmental Science & Technology* **58**, 17916-17925 (2024). https://doi.org/10.1021/acs.est.4c06133

7 Pracht, P., Bohle, F. & Grimme, S. Automated exploration of the low-energy chemical space with fast quantum chemical methods. *Phys. Chem. Chem. Phys.* **22**, 7169-7192 (2020). https://doi.org/10.1039/C9CP06869D

8 Grimme, S., Bannwarth, C. & Shushkov, P. A Robust and Accurate Tight-Binding Quantum Chemical Method for Structures, Vibrational Frequencies, and Noncovalent Interactions of Large Molecular Systems Parametrized for All spd-Block Elements (Z = 1–86). *J. Chem. Theory Comput.* **13**, 1989-2009 (2017). https://doi.org/10.1021/acs.jctc.7b00118

9 Ahlrichs, R., Bär, M., Häser, M., Horn, H. & Kölmel, C. Electronic structure calculations on workstation computers: The program system turbomole. *Chem. Phys. Lett.* **162**, 165-169 (1989). https://doi.org/10.1016/0009-2614(89)85118-8

10 Furche, F. *et al.* Turbomole. *WIREs Comput. Mol. Sci.* **4**, 91-100 (2014). https://doi.org/10.1002/wcms.1162

11 Weigend, F. & Ahlrichs, R. Balanced basis sets of split valence, triple zeta valence and quadruple zeta valence quality for H to Rn: Design and assessment of accuracy. *Phys. Chem. Chem. Phys.* **7**, 3297-3305 (2005). https://doi.org/10.1039/B508541A

12 Becke, A. D. Density-functional exchange-energy approximation with correct asymptotic behavior. *Phys. Rev. A* **38**, 3098-3100 (1988). https://doi.org/10.1103/PhysRevA.38.3098

13 Perdew, J. P. Density-functional approximation for the correlation energy of the inhomogeneous electron gas. *Phys. Rev. B* **33**, 8822-8824 (1986). https://doi.org/10.1103/PhysRevB.33.8822

14 Vosko, S. H., Wilk, L. & Nusair, M. Accurate spin-dependent electron liquid correlation energies for local spin density calculations: a critical analysis. *Can. J. Phys.* **58**, 1200-1211 (1980). https://doi.org/10.1139/p80-159

15 Larriba, C. & Hogan, C. J. Free molecular collision cross section calculation methods for nanoparticles and complex ions with energy accommodation. *J. Comput. Phys.* **251**, 344-363 (2013). https://doi.org/10.1016/j.jcp.2013.05.038

16 Larriba-Andaluz, C. & Hogan, C. J., Jr. Collision cross section calculations for polyatomic ions considering rotating diatomic/linear gas molecules. *J. Chem. Phys.* **141** (2014). https://doi.org/10.1063/1.4901890

17 Weis, P. *et al.* Probing the structure of giant fullerenes by high resolution trapped ion mobility spectrometry. *Phys. Chem. Chem. Phys.* **21**, 18877-18892 (2019). https://doi.org/10.1039/C9CP03326B
